# Effect of 3 Days of Oral Azithromycin on Young Children With Acute Diarrhea in Low-Resource Settings: A Randomized Clinical Trial

**DOI:** 10.1001/jamanetworkopen.2021.36726

**Published:** 2021-12-16

**Authors:** The Antibiotics for Children With Diarrhea (ABCD) Study Group, Tahmeed Ahmed, Mohammod Jobayer Chisti, Muhammad Waliur Rahman, Tahmina Alam, Dilruba Ahmed, Irin Parvin, Md. Farhad Kabir, Sunil Sazawal, Pratibha Dhingra, Arup Dutta, Saikat Deb, Aishwarya Chouhan, Anil Kumar Sharma, Vijay Kumar Jaiswal, Usha Dhingra, Judd L. Walson, Benson O. Singa, Patricia B. Pavlinac, Christine J. McGrath, Churchil Nyabinda, Emily L. Deichsel, Maurine Anyango, Kevin Mwangi Kariuki, Doreen Rwigi, Stephanie N. Tornberg-Belanger, Karen L. Kotloff, Samba O. Sow, Milagritos D. Tapia, Fadima Cheick Haidara, Ashka Mehta, Flanon Coulibaly, Henry Badji, Jasnehta Permala-Booth, Sharon M. Tennant, Dramane Malle, Naor Bar-Zeev, Queen Dube, Bridget Freyne, Nigel Cunliffe, Latif Ndeketa, Desiree Witte, Chifundo Ndamala, Jennifer Cornick, Farah Naz Qamar, Mohammad Tahir Yousafzai, Shahida Qureshi, Sadia Shakoor, Rozina Thobani, Aneeta Hotwani, Furqan Kabir, Jan Mohammed, Karim Manji, Christopher P. Duggan, Rodrick Kisenge, Christopher R. Sudfeld, Upendo Kibwana, Sarah Somji, Mohamed Bakari, Cecylia Msemwa, Abraham Samma, Rajiv Bahl, Ayesha De Costa, Jonathon Simon, Per Ashorn

**Affiliations:** 1 Nutrition and Clinical Services Division, International Centre for Diarrheal Disease Research, Bangladesh, Dhaka, Bangladesh; 2 Laboratory Sciences and Services Division, International Centre for Diarrheal Disease Research, Bangladesh, Dhaka, Bangladesh; 3 Center for Public Health Kinetics, New Delhi, Delhi, India; 4 Childhood Acute Illness and Nutrition Network, Nairobi, Kenya; 5 Department of Global Health, University of Washington, Seattle; 6 Department of Pediatrics, University of Washington, Seattle; 7 Department of Medicine (Allergy and Infectious Diseases), University of Washington, Seattle; 8 Kenya Medical Research Institute, Nairobi, Kenya; 9 Center for Vaccine Development and Global Health, University of Maryland School of Medicine, Baltimore; 10 Department of Epidemiology, University of Washington, Seattle; 11 Department of Pediatrics, Center for Vaccine Development and Global Health, University of Maryland School of Medicine, Baltimore; 12 Department of Medicine, Center for Vaccine Development and Global Health, University of Maryland School of Medicine, Baltimore; 13 Centre pour le Développement des Vaccins, Bamako, Mali; 14 Division of Advanced Primary Health Care Research and Clinical Trials, Centre pour le Développement des Vaccins, Bamako, Mali; 15 Division of Clinical Microbiology and Molecular Biology, Centre pour le Développement des Vaccins, Bamako, Mali; 16 Department of Medicine, Center for Vaccine Development and Global Health, University of Maryland School of Medicine, Baltimore; 17 International Vaccine Access Center, Johns Hopkins Bloomberg School of Public Health, Baltimore, Maryland; 18 Department of Pediatrics, Queen Elizabeth Central Hospital, Blantyre, Malawi; 19 Malawi Liverpool Wellcome Trust Clinical Research Programme, Institute of Infection, Veterinary and Ecological Sciences, The University of Liverpool, Blantyre, Malawi; 20 National Institutes of Health Research Health Protection Research Unit in Gastrointestinal Infections, University of Liverpool, Liverpool, United Kingdom; 21 Malawi Liverpool Wellcome Trust Clinical Research Programme, Liverpool School of Tropical Medicine, Blantyre, Malawi; 22 Malawi Liverpool Wellcome Trust Clinical Research Programme, Blantyre, Malawi; 23 Department of Pediatrics and Child Heath, Aga Khan University, Karachi, Pakistan; 24 Department of Pathology and Laboratory Medicine, Aga Khan University, Karachi, Pakistan; 25 Department of Pediatrics and Child Health, Muhimbili University of Health and Allied Sciences, Dar es Salaam, Tanzania; 26 Division of Gastroenterology, Hepatology and Nutrition, Boston Children’s Hospital, Department of Nutrition, Harvard T.H. Chan School of Public Health, Boston, Massachusetts; 27 Department of Global Health and Population, Harvard T.H. Chan School of Public Health, Boston, Massachusetts; 28 Department of Microbiology and Immunology, Muhimbili University of Health and Allied Sciences, Dar es Salaam, Tanzania; 29 Department of Maternal, Child, and Adolescent Health and Aging, World Health Organization, Geneva, Switzerland

## Abstract

**Importance:**

World Health Organization (WHO) guidelines do not recommend routine antibiotic use for children with acute watery diarrhea. However, recent studies suggest that a significant proportion of such episodes have a bacterial cause and are associated with mortality and growth impairment, especially among children at high risk of diarrhea-associated mortality. Expanding antibiotic use among dehydrated or undernourished children may reduce diarrhea-associated mortality and improve growth.

**Objective:**

To determine whether the addition of azithromycin to standard case management of acute nonbloody watery diarrhea for children aged 2 to 23 months who are dehydrated or undernourished could reduce mortality and improve linear growth.

**Design, Setting, and Participants:**

The Antibiotics for Children with Diarrhea (ABCD) trial was a multicountry, randomized, double-blind, clinical trial among 8266 high-risk children aged 2 to 23 months presenting with acute nonbloody diarrhea. Participants were recruited between July 1, 2017, and July 10, 2019, from 36 outpatient hospital departments or community health centers in a mixture of urban and rural settings in Bangladesh, India, Kenya, Malawi, Mali, Pakistan, and Tanzania. Each participant was followed up for 180 days. Primary analysis included all randomized participants by intention to treat.

**Interventions:**

Enrolled children were randomly assigned to receive either oral azithromycin, 10 mg/kg, or placebo once daily for 3 days in addition to standard WHO case management protocols for the management of acute watery diarrhea.

**Main Outcomes and Measures:**

Primary outcomes included all-cause mortality up to 180 days after enrollment and linear growth faltering 90 days after enrollment.

**Results:**

A total of 8266 children (4463 boys [54.0%]; mean [SD] age, 11.6 [5.3] months) were randomized. A total of 20 of 4133 children in the azithromycin group (0.5%) and 28 of 4135 children in the placebo group (0.7%) died (relative risk, 0.72; 95% CI, 0.40-1.27). The mean (SD) change in length-for-age *z* scores 90 days after enrollment was –0.16 (0.59) in the azithromycin group and −0.19 (0.60) in the placebo group (risk difference, 0.03; 95% CI, 0.01-0.06). Overall mortality was much lower than anticipated, and the trial was stopped for futility at the prespecified interim analysis.

**Conclusions and Relevance:**

The study did not detect a survival benefit for children from the addition of azithromycin to standard WHO case management of acute watery diarrhea in low-resource settings. There was a small reduction in linear growth faltering in the azithromycin group, although the magnitude of this effect was not likely to be clinically significant. In low-resource settings, expansion of antibiotic use is not warranted. Adherence to current WHO case management protocols for watery diarrhea remains appropriate and should be encouraged.

**Trial Registration:**

ClinicalTrials.gov Identifier: NCT03130114

## Key Points

**Question:**

Does the addition of azithromycin to the standard case management of acute watery diarrhea for children aged 2 to 23 months who are dehydrated or undernourished reduce mortality and improve linear growth?

**Findings:**

This randomized clinical trial of 8266 children was unable to detect a survival benefit for children from the addition of azithromycin to the standard World Health Organization (WHO) case management of acute watery diarrhea in low-resource settings.

**Meaning:**

In low-resource settings, adherence to current WHO case management protocols for watery diarrhea remains appropriate; antibiotic use is not warranted.

## Teaser

This randomized clinical trial examines whether the addition of azithromycin to standard case management of acute nonbloody watery diarrhea for children aged 2 to 23 months who are dehydrated or undernourished could reduce mortality and improve linear growth.

## Introduction

Approximately half a million children die annually as a result of acute diarrhea,^1^ mostly in sub-Saharan Africa and south Asia. The current World Health Organization (WHO) guidelines for the case management of acute diarrhea (rehydration, supplemental zinc, continued feeding, and follow-up)^2^ have contributed to significant reductions in diarrhea-associated mortality.^3,4^ These guidelines do not include the use of antibiotics except in the case of bloody diarrhea or suspected cholera.

Studies have shown that 1 or more pathogens can be identified in more than two-thirds of children with acute diarrhea in low- and middle-income settings.^5,6,7^ After rotavirus, bacterial pathogens such as *Shigella*, heat-stable enterotoxin-producing *Escherichia coli* (ST-ETEC), *Campylobacter*, and typical enteropathogenic *E coli* are the leading causes of diarrhea. These bacterial pathogens are associated with subsequent death^8^ and linear growth faltering.^9^ With the implementation of rotavirus vaccine programs, the relative contributions of bacterial causes will likely increase but will remain undiagnosed in the absence of point-of-care diagnostics. Current treatment guidelines may therefore be missing the opportunity to appropriately treat bacterial diarrhea in a select group of young children with dehydrating diarrhea or undernutrition who are at particularly high risk of diarrhea-associated mortality.^1,10,11^

Azithromycin, a macrolide with a broad spectrum of antibacterial activity, is effective against common diarrheal pathogens, including enterotoxigenic *E coli*, *Shigella*, and *Campylobacter* species. In addition, there are yet-uncharacterized survival benefits, as indicated by improved survival among young children who benefitted from the biannual mass administration of drugs in high-mortality settings in Africa.^12,13,14^ At the same time, there are concerns regarding the potential wide use of azithromycin as prophylaxis on the emergence of antimicrobial resistance in children and their communities.

We conducted a multicountry, randomized, placebo-controlled clinical trial (ie, the Antibiotics for Children With Diarrhea [ABCD] trial) to determine whether the addition of azithromycin to the standard case management of acute nonbloody, watery diarrhea among children 2 to 23 months of age who are dehydrated or undernourished could reduce mortality and improve linear growth.

## Methods

### Study Design

The ABCD trial was a multicountry, multicenter, double-blinded, randomized, parallel-group, placebo-controlled clinical trial implemented in Bangladesh, India, Kenya, Malawi, Mali, Pakistan, and Tanzania. The trial protocol has been published^15^ (Supplement 1). Ethics approval was obtained from the WHO Ethics Review Committee as well from the participating countries. Written informed consent was obtained from the primary caregiver. This study followed the Consolidated Standards of Reporting Trials (CONSORT) reporting guideline.

### Study Setting

Participants were recruited between July 1, 2017, and July 10, 2019, from 36 outpatient hospital departments or community health centers in a mixture of urban and rural settings across the 7 countries (eTable 3 in Supplement 2). Staff underwent standardized training in key study processes and in the Integrated Management of Childhood Illness guidelines for the management of acute diarrheal illness.^2^

The 7 countries were selected based on having a large number of children presenting with diarrhea and/or high rates of malnutrition and having a relatively high rate of diarrhea-associated mortality in the Global Enteric Multicenter Study (GEMS),^5^ as well as having teams experienced in the conduct of large intervention trials.

### Participants

#### Screening and Recruitment

All children 2 to 23 months of age presenting with diarrhea at the clinics were screened for inclusion. Children with acute watery diarrhea (≥3 watery stools in the previous 24 hours), some or severe dehydration, and/or moderate wasting (defined as a weight-for-length *z* score >−3 and ≤−2 or a mid–upper arm circumference ≥115 mm and <125 mm) and/or severe stunting were eligible.^15^

Children were excluded if they had dysentery, suspected cholera, severe acute malnutrition, signs of any other infection requiring antibiotic treatment, received antibiotics in the last 14 days, were already enrolled in another trial, or lived outside the study area. Detailed inclusion and exclusion criteria are provided in the trial protocol (Supplement 1).^15^

### Randomization and Masking

Participants were randomized (1:1) to either oral azithromycin, 10 mg/kg/d, once daily for 3 days or placebo. Both active treatment and placebo were supplied as dry sugar-based powders in dark glass bottles. The content of each bottle was identical except for the absence of azithromycin in the placebo.

Site-stratified individual randomization with permuted blocks of varying size (4, 6, or 8) was used. The computer-generated randomization sequence was prepared centrally at the WHO. Allocation was concealed through the use of prelabeled treatment bottles. All sites received identical treatment bottles, labeled by participant number, containing dry powder equivalent to 200 mg/5 mL of azithromycin (1.2 g) or placebo. Participants’ guardians, care providers, and investigators were blinded to randomized group assignment.

Participants received the allocated study treatment immediately after randomization. All doses were administered by research staff in the clinic (day 1) and then administered or directly observed at home (days 2 and 3). For children with some or severe dehydration, randomization was performed only after the child had been stabilized and dehydration was corrected. All participants received standard care for diarrheal disease, including zinc, rehydration, and nutritional counseling following WHO guidelines.^2^

### Study Procedures

Trial personnel collected data at enrollment and at follow-up visits scheduled on days 2, 3, 45, 90, and 180 after enrollment. Participants’ vital status was ascertained through caregiver report at the clinic, at home, and by telephone follow-up visits. Hospital records of participants who were reported to have died were abstracted (if available), and a standardized verbal autopsy interview was conducted to assess the date, cause, and context of death. Hospitalizations were assessed by caregiver report at each study visit. Length, weight, and mid–upper arm circumference were measured at enrollment and at day 90 using standardized procedures.^16^ The WHO Child Growth Standards were used to calculate sex- and age-standardized *z* scores.^17^ Stool and nasopharyngeal samples were collected on day 1 (stool from participants only), day 90, and day 180 from participants and a household child contact (sibling) for isolation of *E coli* and *Streptococcus pneumoniae*, respectively. Sensitivity to azithromycin was tested using the E-test^18^ for *E coli* and the Microscan Autoscan4 (Beckman Coulter) for *S pneumoniae*. Sensitivity to other antibiotics was tested using the Microscan Autoscan4. Clinical minimum inhibitory concentration cutoffs corresponding to nonsusceptibility from the 2016 Clinical and Laboratory Standards Institute^19^ were used to define resistant isolates of *E coli* and *S pneumoniae.*

### Study Outcomes

Two primary outcomes were compared by trial group: (1) all-cause mortality within 180 days of enrollment and (2) change in linear growth measured as change in the length-for-age *z* score between enrollment and day 90 after enrollment. Secondary outcomes included change in anthropometric measures of acute malnutrition (weight-for-age *z* score, weight-for-length *z* score, and mid–upper arm circumference), proportion of children hospitalized in the 90 days after enrollment, and the composite proportion of children hospitalized or who had died 10 days after enrollment or 90 days after enrollment. Furthermore, the prevalence of antimicrobial resistance to azithromycin among *E coli* and *S pneumoniae* isolates at day 90 and day 180 was compared by group in a random subsample of children enrolled in the ABCD trial in year 1 and their household contacts (siblings).

Definitions of all outcomes are provided in the protocol and statistical analysis plan (Supplement 1).^20,21^ All participant-related information was stored securely at study sites. Data were entered into a web-based, data management platform and centrally managed by RTI International.

### Statistical Analysis

Based on previous data from GEMS,^5^ we anticipated 2.7% mortality by day 180 in the control group. At 1:1 allocation, 5750 children per group would provide 90% power at 95% confidence of detecting a 35% or more reduction in mortality in the azithromycin group, allowing for 10% loss to follow-up before completion and 1 interim analysis. This size provided 80% power and 95% confidence to detect a 0.04 or more absolute difference in the mean change in the length-for-age *z* score between study groups at day 90, assuming an SD of 0.7. All *P* values were from 2-sided tests and results were deemed statistically significant at *P* < .05.

Analytical plans were defined a priori and have been published.^21^ Primary analysis included all randomized participants by intention to treat. To obtain a relative risk for death up to day 180, a log-binomial regression (including country as a covariate to account for randomization scheme) was performed. Censored individuals who withdrew consent or were lost to follow-up were considered to be living. For the coprimary outcome, we used a linear regression model adjusted for country and baseline length-for-age *z* score to compare participants surviving to day 90 in the 2 groups. We also conducted prespecified subgroup analyses for the primary outcomes by site, age, sex, anthropometry, and socioeconomic status. Outcomes were not adjusted for multiple comparisons. For comparisons of antimicrobial resistance between groups, a noninferiority margin of 10% (absolute difference) was defined and tested by the continuity-corrected χ^2^ test.^22^

Accruing data were monitored, in confidence, by the data safety monitoring board (DSMB) with an interim analysis performed in June 2019, when 40% and 50% of the proposed 11 500 children had been followed up for the primary mortality outcome and the growth outcome, respectively. The DSMB recommended that the trial be stopped for futility on the mortality outcome because available data indicated that the predictive power for rejecting the null hypothesis of no difference in mortality by day 180 was 7% if the trial continued recruitment to target enrollment. For change in length-for-age *z* score, despite a reasonable predictive power (68%) to reject the null hypothesis of no difference in change in length-for-age *z* score, the DSMB considered the effect size to be of insufficient clinical or public health relevance significance. Recruitment was stopped at all sites thereafter, although all participants recruited up to that point were followed up for 180 days from recruitment. All ethics committees and regulatory authorities were informed.

## Results

Of 66 379 children screened for eligibility, 8268 were randomized (4463 boys [54.0%]; mean [SD] age, 11.6 [5.3] months; 4133 to the azithromycin group and 4135 to the placebo group) from July 1, 2017, through July 10, 2019 (Table 1; Figure). A total of 2726 children (33.0%) had moderate wasting, and 1249 (15.1%) had severe stunting (Table 1). The most common reasons for ineligibility were either the absence of dehydration or undernutrition or the receipt of antibiotics in the 14 days prior to screening. There were no differences between groups in demographic and anthropometric characteristics at baseline. We had complete data on 3934 of 4135 children (95.1%) in the placebo group and 3920 of 4133 children (94.8%) in the azithromycin group.

**Table 1.** Characteristics of Participants at Baseline

Characteristic	Mean (SD)	
	Placebo group (n = 4135)	Azithromycin group (n = 4133)
Age, mo	11.6 (5.3)	11.7 (5.2)
Sex		
Male	2219 (53.7)	2244 (54.3)
Female	1916 (46.3)	1889 (45.7)
Anthropometry		
Weight, kg	7.5 (1.5)	7.5 (1.4)
Length, cm	70.0 (6.4)	70.2 (6.2)
MUAC, cm	13.1 (1.21)	13.1 (1.2)
LAZ, *z* score	–1.5 (1.3)	–1.5 (1.3)
WLZ, *z* score	–1.1 (1.2)	–1.1 (1.2)
Prevalence of severe stunting (LAZ ≤−3.0), No. (%)	614 (14.8)	635 (15.4)
Prevalence of moderate wasting (−3.0 <WLZ ≤−2.0), No. (%)	1340 (32.4)	1386 (33.5)
Prevalence of some or severe dehydration, No. (%)	2278 (55.1)	2232 (54.0)
Maternal characteristics		
Age, y	25.7 (5.6)	25.7 (5.6)
Height, cm	155.2 (7.3)	155.2 (7.6)
Weight, kg	56.2 (12.4)	56.1 (12.3)
BMI	23.3 (4.5)	23.2 (4.7)
Education, y	6.1 (4.3)	6.1 (4.4)
No. of children <5 y in the household	1.6 (0.9)	1.7 (1.1)
Socioeconomic wealth quintile^a^	3.3 (1.4)	3.5 (1.4)
Azithromycin-resistant *Escherichia coli* in stool, No./total No. (%)	257/958 (26.8)	264/933 (28.3)

Abbreviations: BMI, body mass index (calculated as weight in kilograms divided by height in meters squared); LAZ, length-for-age *z* score; MUAC, mid–upper arm circumference; WLZ, weight-for-length *z* score.

^a^ Wealth quintiles constructed against country-specific wealth distributions as reported in each country’s most recent Demographic and Health Survey.

**Figure.**
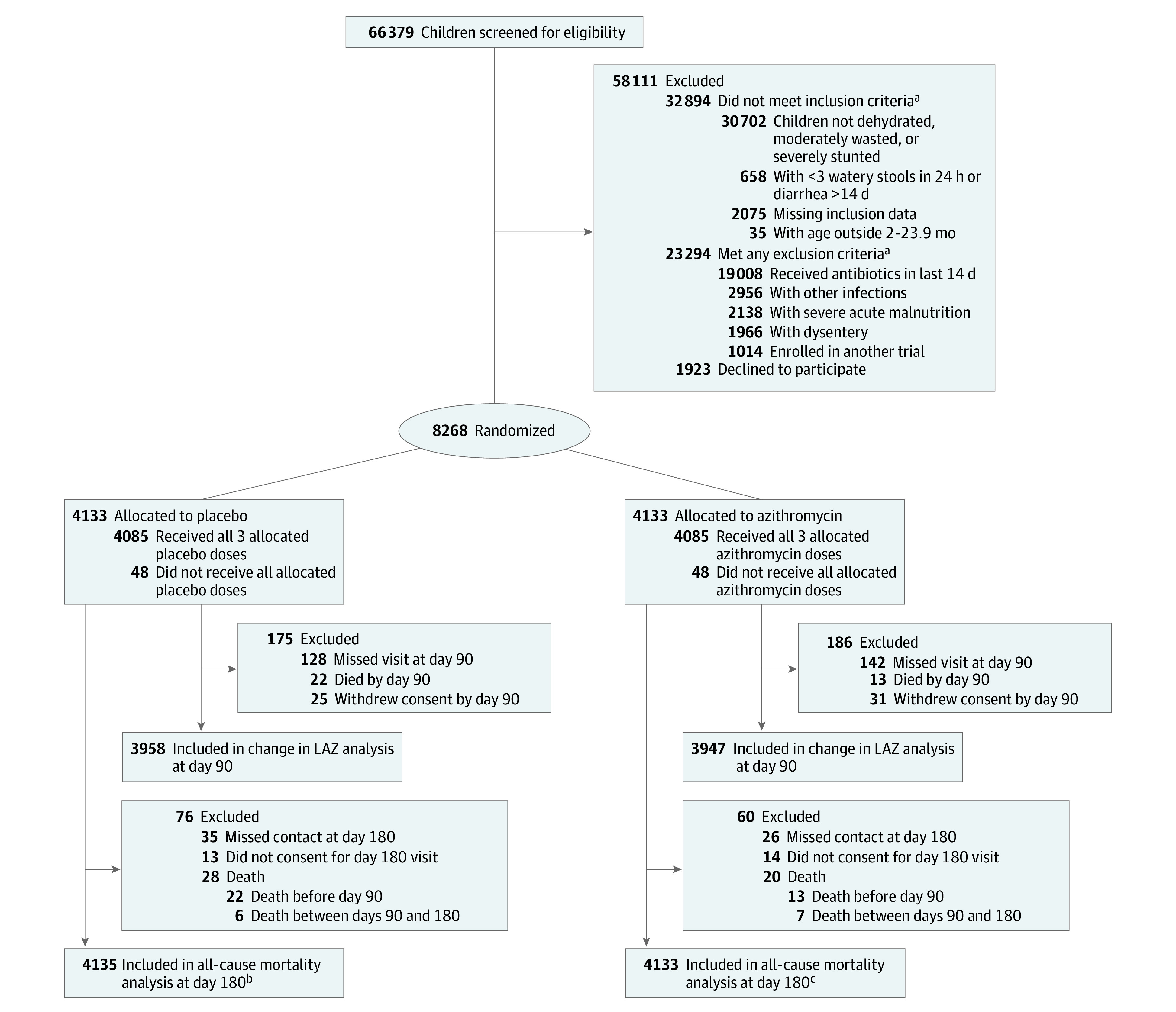
Study Flowchart LAZ indicates length-for-age *z* score. ^a^Children fell into more than 1 of the following categories. ^b^Outcome (vital status) available for 4087 children in the placebo group; 48 additional children were presumed alive. ^c^Outcome (vital status) available for 4093 children in the placebo group; 40 additional children were presumed alive.

Overall, 52 of 4133 children (1.3%) in the azithromycin group and 48 of 4135 children (1.2%) in the placebo group did not receive all 3 doses of the treatment. There were 20 deaths (0.5%) in the azithromycin group and 28 deaths (0.7%) in the placebo group (relative risk, 0.72; 95% CI, 0.40-1.27; *P* = .25) (Table 2). The mean (SD) change in length-for-age *z* score from day 1 to day 90 was −0.16 (0.59) in the azithromycin group and −0.19 (0.60) in the placebo group (risk difference, 0.03; 95% CI, 0.01-0.06; *P* = .007). The results were similar in a per-protocol analysis (eTable 2 in Supplement 2).

**Table 2.** Primary and Secondary Outcomes

Outcome	Placebo group (n = 4135)	Azithromycin group (n = 4133)	RR or RD (95% CI)	*P* value
Primary outcomes				
180-d Mortality, No. (%)^a^	28 (0.7)	20 (0.5)	RR, 0.72 (0.40 to 1.27)^b^	.25
90-d ΔLAZ, change, mean (SD)	–0.19 (0.60)	–0.16 (0.59)	RD, 0.03 (0.01 to 0.06)^c^	.007
Secondary outcomes, No. (%)				
Hospitalization or death^d^				
By day 10	68 (1.6)	45 (1.1)	RR, 0.66 (0.46 to 0.96)	.31
By day 90	226 (5.5)	178 (4.3)	RR, 0.79 (0.65 to 0.95)	.01
Hospitalization				
By day 90	211 (5.1)	170 (4.1)	RR, 0.81 (0.66 to 0.98)	.03
Anthropometric outcomes, change day 1-90, mean (SD)^a^^,^^e^				
ΔWAZ	0.17 (0.57)	0.20 (0.57)	RD, 0.02 (0.00 to 0.05)	.05
ΔWLZ	0.28 (0.82)	0.30 (0.82)	RD, 0.01 (−0.02 to 0.05)	.43
ΔMUAC	0.60 (0.71)	0.61 (0.68)	RD, 0.00 (−0.03 to 0.03)	.98

Abbreviations: ΔLAZ, change in length-for-age *z* score; ΔMUAC, change in mid–upper arm circumference; ΔWAZ, change in weight-for-age *z* score; ΔWLZ, change in weight-for-length *z* score; RD, risk difference; RR, relative risk.

^a^ Causes of death (eTable 1 in Supplement 2).

^b^ Relative risk from log-binomial regression, adjusted for country.

^c^ Risk difference in ΔLAZ from linear regression adjusted for baseline LAZ and country.

^d^ Log-binomial regression adjusted for country, effect in terms of relative risk, 95% CI.

^e^ Linear regression, respectively, of WAZ, WLZ, and MUAC adjusted for baseline WAZ, WLZ, and MUAC and country.

No evidence of modification of treatment effect with respect to participant age, sex, wealth quintile, or reason for enrollment was noted for either primary outcome (eFigures 1 and 2 in Supplement 2). For the combined outcome of death or hospitalization, there were 45 events by day 10 in the azithromycin group and 68 events by day 10 in the placebo group (1.1% vs 1.6%; relative risk, 0.66; 95% CI, 0.46-0.96) (Table 2). At day 90, there were 178 events in the azithromycin group and 226 events in the placebo group (4.3% vs 5.5%; relative risk, 0.79; 95% CI, 0.65-0.95). There were 170 hospitalizations by day 90 in the azithromycin group compared with 211 hospitalizations in the placebo group (4.1% vs 5.1%; relative risk, 0.81; 95% CI, 0.66-0.98). There were no statistically significant differences in the change in other anthropometric indices between the 2 groups.

Resistance to azithromycin in stool *E coli* and in nasopharyngeal *S pneumoniae* from participants in the azithromycin group was not greater than that in the placebo group at both day 90 (207 of 848 [24.4%] vs 213 of 875 [24.3%] for *E coli* and 238 of 838 [28.4%] vs 227 of 869 [26.1%] for *S pneumoniae*) and day 180 (186 of 805 [23.1%] vs 177 of 846 [20.9%] for *E coli* and 211 of 783 [26.9%] vs 217 of 826 [26.3%] for *S pneumoniae*), using noninferiority analysis (Table 3). Similarly, resistance to azithromycin in stool *E coli* and in nasopharyngeal *S pneumoniae* from household contacts of participants in the treatment group was not greater than that in the placebo group at both day 90 (52 of 372 [14.0%] vs 48 of 385 [12.5%] for *E coli* and 87 of 372 [23.4%] vs 81 of 385 [21.0%] for *S pneumoniae*) and day 180 (47 of 333 [14.1%] vs 50/358 [14.0%] for *E coli* and 63 of 333 [18.9%] vs 75 of 359 [20.9%] for *S pneumoniae*).

**Table 3.** Antimicrobial Resistance to Azithromycin in Participant Children and Their Household Contacts^a^

Characteristic	No./total No. (%)		Risk difference (95% CI)^b^
	Placebo group	Azithromycin group	
**Participants^c^**			
*Escherichia coli*			
Day 90	213/875 (24.3)	207/848 (24.4)	0.001 (−0.04 to 0.04)
Day 180	177/846 (20.9)	186/805 (23.1)	0.02 (−0.02 to 0.06)
*Streptococcus pneumoniae*			
Day 90	227/869 (26.1)	238/838 (28.4)	0.02 (−0.02 to 0.06)
Day 180	217/826 (26.3)	211/783 (26.9)	0.01 (−0.04 to 0.05)
**Contacts**			
*Escherichia coli*			
Day 90	48/385 (12.5)	52/372 (14.0)	0.02 (−0.03 to 0.06)
Day 180	50/358 (14.0)	47/333 (14.1)	0.001 (−0.05 to 0.05)
*Streptococcus pneumoniae*			
Day 90	81/385 (21.0)	87/372 (23.4)	0.02 (−0.04 to 0.08)
Day 180	75/359 (20.9)	63/333 (18.9)	−0.02 (−0.08 to 0.04)

^a^ Resistance: intermediate and resistant samples were considered as resistant.

^b^ Noninferiority: resistance in the azithromycin group will not exceed resistance in placebo group by more than 10% (noninferiority margin). Adjusted by country.

^c^ A total of 1896 participant children were enrolled in the antimicrobial resistance substudy.

There were no differences between the 2 groups with regard to resistance shown by *E coli* or *S pneumoniae* isolates to other classes of antibiotics at any of the time points tested up to day 180 in either index children or their contacts (eTables 4-7 in Supplement 2).

### Serious Adverse Events

Hospitalization or death by day 10 was considered to be a serious adverse event for this study; this outcome was statistically significantly lower in the azithromycin group than in the placebo group (45 of 4133 [1.1%] and 68 of 4135 [1.6%]) (Table 2). Two other serious events, seizures (azithromycin group) and severe colitis (placebo group), occurred in 1 participant each.

## Discussion

In this large, multicenter randomized clinical trial, we did not detect a difference in mortality within 180 days of enrollment between children who received azithromycin and those who received placebo, in addition to standard WHO treatment for acute watery diarrhea. A small statistically significant difference between groups was observed for the coprimary outcome of change in linear growth (change in length-for-age *z* score) between enrollment and day 90. This small difference corresponds to a 1-mm difference in length over 3 months for a 1-year-old child, a change unlikely to be of clinical or public health significance.

We found that 180-day mortality in the control group (0.7%) was substantially lower in our study than the mortality assumption used in sample size estimations (2.7%), which were based on GEMS.^5^ It is likely that differences between the GEMS and ABCD trial populations with regard to (1) eligibility criteria related to the severity of the diarrheal illness, (2) the degree of malnutrition, and (3) the rigor of management protocols (GEMS was an observational study in routine care settings, whereas ABCD was a trial in which strict protocols following the WHO guidelines for the management of diarrhea were adhered to), as well as global trends of decreasing diarrheal disease mortality, contributed to the differences between projected and observed mortality rates.

Although the observed mortality rate was slightly lower in the azithromycin group (0.5%) compared with the placebo group (0.7%), the number of children required to detect this difference with adequate statistical power would be 3 times higher than originally planned. This led the DSMB to stop trial recruitment early for futility.

Although the effect of azithromycin on cholera^23,24^ and salmonella enteritis^25^ has been studied, the effect of azithromycin on mortality among children with acute diarrhea has not been reported, to our knowledge. Mass administration of azithromycin has been shown to reduce overall mortality, but only in high-mortality settings and among children younger than 6 months.^12,13^ Although the mechanism of action of improved survival in mass drug administration trials remains unknown, post hoc subanalyses examining whether prevention of diarrhea and dysentery could have played a role are inconclusive.^26^ Secondary analysis of a trial of the mass administration of seasonal malaria prophylaxis plus azithromycin or placebo^27^ showed no evidence of a protective effect of azithromycin on hospitalization and deaths, and protection from infectious illnesses was short-lived (2-4 weeks).^28^ A trial in Niger is under way to test the effect of age-based targeting of biannual azithromycin on mortality and antimicrobial resistance in children.^29^

Although transient increases in weight gain have been reported among children younger than 5 years who received azithromycin compared with placebo,^30^ previous studies have not found a substantial benefit of azithromycin for linear growth.^31,32,33^ A pilot trial in Burkina Faso testing the efficacy of azithromycin to improve nutritional outcomes in children with uncomplicated severe acute malnutrition has recently completed recruitment.^34^

With regard to secondary outcomes, the difference in the composite outcome of hospitalization or death merits consideration. There was a 21% lower risk of hospitalization or death in the 90-day follow-up period in the azithromycin group. Similar results were observed for this outcome in the first 10 days of follow-up. At both time points, this difference was driven largely by hospitalization, defined as any overnight stay in the hospital regardless of illness severity. Because hospitalization is dependent on care seeking and severity of illness, randomization and double blinding make it less likely that care seeking may have been different by trial groups. Nevertheless, admission practice is likely to vary substantially by context; 80% of hospitalizations were in the Asian sites included in our study. Thus, the public health and clinical relevance of this finding needs to be interpreted with caution given that the reason for hospitalization or severity of illness that prompted hospitalization was not routinely documented, and that no adjustment was made for multiple comparisons on the secondary outcomes. This finding needs to be confirmed in future research in different contexts of region and childhood illness.

There were no differences in the other anthropometric indices between groups. There was no difference in the proportion of azithromycin resistance in *E coli* and *S pneumoniae* between children who received azithromycin and children who received placebo, nor among their household contacts. This finding contrasts with those from studies on mass administration of azithromycin for control of trachoma^35^ or for improving child survival,^36^ both of which showed that mass drug administration was associated with carriage of macrolide-resistant *E coli*. However, both of these studies involved repeated cycles of mass administration of azithromycin.

### Strengths and Limitations

This study has several strengths. This was a double-blind, randomized, placebo-controlled clinical study, which was conducted using standardized intervention delivery and outcome measurement procedures, thus minimizing selection and measurement bias. The sample size was large, and the study was conducted in 3 countries in south Asia and 4 countries in sub-Saharan Africa, making the findings relevant to settings with the largest burden of childhood diarrhea deaths. Loss to follow-up was low (5%).

This study also has some limitations. This trial can neither demonstrate nor exclude the possibility of a small difference in mortality associated with azithromycin because of the low risk of mortality in the control group. We would have needed a study that was at least 3 times as large to have 80% power to detect the observed difference in mortality. Although trial participants are representative of children who present with diarrhea to first-level health facilities in low- and middle-income countries, we did not include those with more severe diarrheal disease or severe malnutrition.

The results of this trial have programmatic implications. Mortality in this trial was low, although the children presenting with diarrhea who enrolled in the trial were vulnerable given their age, dehydration status, and/or the presence of underlying malnutrition. We hypothesize that the trial requirement for rigorous adherence to the WHO guidelines for the management of diarrhea^2^ substantially contributed to the low observed mortality.^4^ Household follow-up visits to directly administer or observe treatment administration may have also had an effect on observed mortality owing to the Hawthorne effect. Although these data do not support the addition of azithromycin to standard care for diarrheal diseases in these settings, they do suggest prioritizing resources for diarrheal disease treatment in accordance with WHO guidelines and follow-up for high-risk children.

## Conclusions

The ABCD trial did not detect an improvement in survival from the addition of azithromycin to standard WHO case management of acute watery diarrhea. Although a small reduction in linear growth faltering was seen in the treatment group, this is unlikely to be of clinical or public health significance. These results support rigorous adherence to the current WHO guidelines on the management of diarrhea and do not suggest that a change to global diarrhea management guidelines or policy is merited.

## Notes

Supplement 1.
Trial Protocol

Supplement 2. **eTable 1.** Cause of Death Among Children in the ABCD Trial **eTable 2.** Per Protocol Analyses of Primary Outcomes **eTable 3.** List of Recruiting Centres **eTable 4.** Proportion of *E. coli* Isolates From Index Children With Antibiotic Resistance by Randomization Arm and Timepoint **eTable 5.** Proportion of *S. pneumoniae* Isolates From Index Children With Antibiotic Resistance by Randomization Arm and Timepoint **eTable 6.** Proportion of *E. coli* Isolates From Child Contacts With Antibiotic Resistance by Randomization Arm and Timepoint **eTable 7.** Proportion of *S. pneumoniae* Isolates From Child Contacts With Antibiotic Resistance by Randomization Arm and Timepoint **eFigure 1.** Effect Modification on the Primary Outcome (Mortality) in Different Strata (Point Estimates and 95% CI) by a Priori Defined Effect Modifiers **eFigure 2.** Effect Modification on the Primary Outcome (Change in Linear Growth) in Different Strata (Point Estimates and 95% CI) by a Priori Defined Effect Modifiers

Supplement 3.
Data Sharing Statement
