# Supplementary material for: Effect of 3 Days of Oral Azithromycin on Young Children With Acute Diarrhea in Low-Resource Settings: A Randomized Clinical Trial
Source: JAMA Netw Open. 2021 Dec 16;4(12):e2136726. doi: 10.1001/jamanetworkopen.2021.36726 (PMC8678692; doi:10.1001/jamanetworkopen.2021.36726)
Supplement: Supplement 1. — Trial Protocol [file jamanetwopen-e2136726-s001.pdf]

Study protocol: Antibiotics for Children with Severe Diarrhoea (ABCD) Trial

Overall Study Coordination

World Health Organization, Maternal Newborn, Child and Adolescent Health Research and Development Team, Geneva, Switzerland

Field Sites

Centre pour le Développement des Vaccins (CVD-Mali), Bamako, Mali
Kenya Medical Research Institute (KEMRI)
Muhimbili University of Health and Allied Sciences, Dar es Salaam, Tanzania
Malawi-Liverpool-Wellcome Trust Clinical Research Programme, Blantyre, Malawi
International centre for diarrhoeal disease research, Bangladesh (icddr,b), Dhaka, Bangladesh
Centre for Public Health Kinetics, Delhi, India
Aga Khan University (AKU), Karachi, Pakistan

WHO Coordinators: Dr. Jonathon Simon, Dr. Rajiv Bahl

Principal Investigators:

Bangladesh
Dr Tahmeed Ahmed (icddr,b, Dhaka, Bangladesh)
Dr Md. Jobayer Chisti (icddr,b, Dhaka, Bangladesh)

India
Dr Sunil Sawazal (Centre for Public Health Kinetics, Delhi, India)
Dr. Usha Dhingra (Centre for Public Health Kinetics, Delhi, India)

Kenya
Dr. Benson Singa (Kenya Medical Research Institute, Nairobi, KE)
Dr Judd L. Walson (University of Washington, Seattle, WA, USA)

Malawi
Dr Queen Dube (Queen Elizabeth Central Hospital, Blantyre, Malawi)
Dr Naor Bar-Zeev (Johns Hopkins University, Baltimore, MD, USA and University of Liverpool, Liverpool, UK and Malawi-Liverpool-Wellcome Trust Clinical Research Programme, Blantyre, Malawi)

Mali
Dr Samba O Sow (CVD-Mali, CNAM, Bamako, Mali)
Dr Karen L Kotloff (University of Maryland, Baltimore, MD, USA)

Pakistan
Dr. Farah Qamar (AKU, Karachi, Pakistan)
Dr. Tahir Yousafzai (AKU, Karachi, Pakistan)

Tanzania
Dr Karim Manji (Muhimbili University of Health and Allied Sciences, Dar es Salaam, TZ)
Dr Chris Duggan (Boston Children's Hospital, Boston, MA, USA)

TABLE OF CONTENTS

PROTOCOL VERSIONS AND AMENDMENT HISTORY 5

0. BACKGROUND 6

1. OBJECTIVES 9

1.1 Co-Primary Objectives: 9

1.2 Secondary Objectives: 10

2. SIGNIFICANCE AND IMPLICATIONS 10

2.1 Study sites 11

2.2 Study Design 11

2.3 Eligibility criteria 11

2.4 Intervention 12

2.5 Duration of follow-up 12

3. RATIONALE FOR THE DEFINITION OF STUDY POPULATION AND ELIGIBILITY CRITERIA 13

3.1 Study Population 13

3.2 Inclusion criteria 13

3.3 Exclusion criteria 13

4. RECRUITMENT 14

4.1 Screening 14

4.2 Stabilization period 14

4.3 Consent 14

4.4 Enrolment 14

5. STANDARD MANAGEMENT AND INTERVENTION 15

5.1 Study Drugs Intervention 15

5.2 Randomization and blinding 15

5.3 Study drugs administration 16

6. FOLLOW UP 17

6.1 Day 45 Follow up 17

6.2 Day 90 Follow up 17

6.3 Day 180 Follow up 18

6.4 Outcome measurements 18

6.5 Collection and processing of biological samples 18

7. SERIOUS ADVERSE EVENTS 20

8. TRAINING **21**
9. TIMELINE **21**
10. DATA ANALYSIS **21**
10.1 Primary Objectives 22
10.2 Secondary Objectives 23
10.3 Sample size 25
11. RISK MITIGATION **27**
12 STUDY MANAGEMENT AND OVERSIGHT **29**
12.1 Coordination 29
12.2 Local Governance 29
12.3 Ethical Review 30
12.4 Trial Advisory Group and Technical Steering Committee 30
12.5 Data Safety and Monitoring Board 30
12.6 Quality assurance 30
12.7 Data management 31
12.8 References 32
13 SITE IMPLEMENTATION PLANS **33**
13.1 Bangladesh 33
13.2 India 37
13.3 Kenya 40
13.4 Malawi 44
13.5 Mali 47
13.6 Pakistan 50
13.7 Tanzania 53

STUDY OVERVIEW

Although the current WHO recommended management package for acute diarrhoea (ORS, zinc and feeding advice) has contributed to significant reductions in diarrhoea associated mortality, over half a million children continue to die annually as a result of acute diarrhoeal episodes. In addition, there is increasing interest in the rates of mortality in young children in the 90-180 days following an episode of acute diarrhoea. The long-term benefits of antibiotic administration may result from direct antimicrobial effects on pathogens or from other incompletely understood mechanisms including improved nutrition, alterations in immune tolerance or improved enteric function. Optimizing antibiotic treatment of acute diarrhoea episodes in very young children with severe disease may offer the opportunity to significantly reduce diarrhoea associated deaths in the 90-180 days following presentation for acute diarrhoea and may also improve growth. We propose to evaluate the efficacy of an antibiotic (azithromycin) delivered in a specific, targeted fashion to young children (< 2 years of age) at high risk of diarrhoea associated mortality in a multi-site randomized, double-blind, placebo-controlled trial. The study will evaluate the ability of the intervention to reduce mortality within 180 days of the acute diarrhoeal episode and improve nutritional status over the same period.

PROTOCOL VERSIONS AND AMENDMENT HISTORY

Version	Date issued	Authors	Details of changes made	WHO ERC submission & status; dates and number
1.0	05.02. 2016	Simon, Bahl	Original document, submitted to the WHO ERC	Submitted 10.02.-16, conditionally approved 10.03.-16 (ERC.0002722)
2.0			Internal working document, not submitted to IRB	
3.0	01.05. 2016	Simon, Bahl	Provided clarifications based on comments by the WHO. No changes to study design	Submitted 09.05.-16, approved 14.07.-16 (pending site-specific protocols)
4.0			Internal working document, not submitted to IRB	
5.0			Internal working document, not submitted to IRB	
6.0	08.02. 2017	Ashorn, Simon, Bahl	Removed ciprofloxacin from study interventions; because of concerns about the safety of this antibiotic and a recommendation by a WHO expert group. Accordingly, removed “allergy to ciprofloxacin” from exclusion criteria, changed sample size, and edited analysis strategy to match the new 2-arm (vs. earlier 3-arm) design<br>Tightened inclusion criteria so that children with moderate stunting will not be enrolled, because of concerns about spread of antimicrobial resistance and recommendation by the WHO expert group: Relatedly, clarified objectives and methods for biological specimen collection and processing for analysis of bacterial resistance to antimicrobial agents.<br>Added number and cause of hospitalizations in outcome measures, based on requests from some local IRBs<br>Corrected some small errors in the previous version: Edited mid upper arm circumference (MUAC) cut-off to determine severe wasting in exclusion criteria, to match WHO and national criteria (from MUAC <110 mm to MUAC <115 mm); removed “visible wasting” from exclusion criteria (not operational); Added “participation in another interventional trial” as an exclusion criterion; Clarified the definition of some eligibility criteria<br>Made some technical edits to the document: Clarified the role and meeting modes of Trial Advisory Group, Trial Steering Committee, and Data Safety and Monitoring Board; Added a cover page with list of investigators, a table of contents and a table explaining the version history and edits to the protocol; Edited informed consent document to reflect the changes in the study design; Added an informed consent document to be used for a guardian interview on the cause of death if a study participant dies	Submitted 08.02.-17, clarification sought 24.02.2017
7.0	01.03. 2017	Ashorn, Simon, Bahl	Clarified the storage and analysis of biological specimens, edited informed consent on the same.	Submitted 01.03.-17 Approved 07.03.2017
8.0	30.10.	Ashorn,	Added a possibility to collect stool samples by rectal	To be submitted to the

Version	Date issued	Authors	Details of changes made	WHO ERC submission & status; dates and number
	2017	Simon, Bahl	swabs. Added a mother as a possible household contact from whom a nasopharyngeal swab can be collected for antimicrobial resistance analysis. Added a follow-up visit to day 180 for all participants. Corrected a typo in participant numbers in Figure 1. Edited informed consent on changes	Submitted 02.03.-18<br>Approved 25.03.2018
9.0	21.12.2018	Simon	Modified Secondary Outcomes<br>Modified Analysis Plan based on the secondary outcomes changes<br>Removed Dr. Per Ashorn as WHO Coordinator<br>Adjusted sample size for AMR component	Submitted to the WHO ERC after site approvals of the modification

0. BACKGROUND

The problem

Despite dramatic reductions in childhood mortality during the past three decades, an estimated 6.3 million children died in 2013 before reaching their 5th birthday. The reduction in mortality has been especially impressive for diarrhoeal disease. In 1980, an estimated 4.6 million under-five children were dying every year because of diarrhoea (2) while in 2013, the number of deaths due to diarrhoea in this age group was reduced to 0.6 million per year (3). This very significant reduction was partly due to the diarrhoea control strategy implemented throughout the world since the 1980s which includes the treatment/prevention of dehydration with ORS and/or IV fluids, administration of zinc, and feeding advice.

There continue to be over 600,000 deaths/year in under-five children attributed directly to diarrhoea. Data from the Global Enterics Multicenter Study (GEMS), a large multi-center observational study, suggest that only 1/3 of diarrhoea associated deaths occur in the first week following diagnosis. When follow-up visits were conducted 50-90 days after the enrolment encounter, the timing of deaths among children with diarrhoea was as follows: 34% occurred on days 0–7 after enrolment, 33% on days 8–21 and 33% after day 21 (4). In addition, diarrhoea episodes considered as moderate to severe cases had an 8.5fold higher risk of death during the 50-90 day follow-up period compared to their matched controls in spite of appropriate treatment of the episode, including ORS, zinc and feeding advice (4). The risk of death was significantly associated with the degree of stunting at enrolment. Although 26% of deaths in cases occurred during the enrolment encounter at the health care facility and 19% during a subsequent medical contact, importantly, 55% occurred at home or outside of a medical facility. Most deaths in patients with diarrhoea occurred in infants (56%) and toddlers (32%). These data suggest that current estimates which predominantly capture immediate deaths and those that occur in a health facility may dramatically underestimate diarrhoea associated mortality.

These data also suggest that a proportion of deaths due to diarrhoea, especially in young children and in children with moderate to severe diarrhoea, may not be avoided just by using

the current treatment recommendations for ORS, zinc and continued feeding. This is confirmed by a recently conducted verbal autopsy study which showed that most children (70%) who died from diarrhoea had received ORS or intravenous solutions (5) prior to death. Additional treatment may be necessary in these children to reduce diarrhoea mortality.

Potential Solution:

The World Health Organization currently recommends the use of antibiotics only for the treatment of dysentery (as a proxy for suspected shigellosis and cholera) (6, 7). However, recent data suggest (4) that multiple bacterial and parasitic pathogens are important in the aetiology of moderate to severe diarrhoea and some of these pathogens are significantly associated with death in the 90 days following a diarrhoea episode (Cryptosporidium, typical enteropathogenic E. coli (tEPEC) and ST-ETEC).

Given the demonstrated risk of death associated with diarrhoea in specific subsets of children with malnutrition and those with severe illness at baseline, expanding the use of antibiotics for a subset of children with diarrhoea associated with high risk of subsequent mortality (high risk diarrhoea) may be an important intervention to reduce diarrhoea associated mortality.

Recently published data from the GEMS study suggest that least one putative pathogen can be identified in over 80% of children presenting with moderate-severe diarrhoea. In addition to viruses (including rotavirus), bacterial pathogens, including Shigella and pathogenic E. coli species were commonly identified across all seven of the sites, all with moderate-to-high under-5 mortality in sub-Saharan Africa and South Asia (4). Other authors have also demonstrated that visible blood in stool is a poor indicator of Shigella infection in children with diarrhoea (Pavlinac, ASTMH 2014). Taken together, these data suggest that current treatment guidelines may be missing the opportunity to appropriately provide antibiotics to a group of young children at particularly high risk of diarrhoea associated mortality.

Data suggest that 80-90% of all children with diarrhoea currently receive antibiotics, despite WHO guidelines restricting antibiotics to a small subset with dysentery or cholera. The use of targeted empiric antibiotics for a small subgroup of severely ill children at high risk of death may also potentially lower antimicrobial resistance if such children defer to the use of more appropriate antibiotics than are commonly used in practice now (despite WHO guidelines) and if other children are less likely to use antibiotics based on an assessment of their being at lower risk. In addition, data from trials of antibiotics in acute respiratory illness also suggest that when clear guidelines are provided for the use of antibiotics in high risk children, antibiotic use decreases and resistance among isolated pathogens is reduced (10, 11).

After discussions with antimicrobial resistance experts at WHO and externally, we feel that careful documentation of the association between clinical failure and the development of resistance in the community during this trial would be useful to allow careful risk/benefit assessment of any proposed policy change if a mortality reduction benefit is shown. In a complementary linked study, we propose to study antimicrobial resistance to multiple antibiotics, among *Escherichia coli* and *Streptococcus pneumonia* isolates in stool samples collected from a subgroup of participants at enrolment and in stool samples and naso-

pharyngeal swabs collected from the same subgroup of participants and their close household contacts at the 90-day and 180 days follow-ups. Additionally, we propose that stool be collected and stored at -80°C from all participants at enrolment for molecular detection of pathogens and identification of antimicrobial resistance genes at a later date. For the same purpose, we also propose to store stool and nasopharyngeal swab samples from the subgroup of participants at the 90-day and 180-day follow-ups.

Safety profile of azithromycin: A review of studies having used azithromycin in young children concluded that "…azithromycin given as a suspension once daily for 3 days is well tolerated and can be used safely for the treatment of acute bacterial infections in children of all ages. Even in very young children, there are no concerns regarding safety or tolerance." (13).

References

1. Liu L, Johnson HL, Cousens S, et al. Global, regional, and national causes of child mortality: an updated systematic analysis for 2010 with time trends since 2000. Lancet, 2012; 379:2151-2161.

2. Snyder JD, Merson MH. The magnitude of the global problem of acute diarrhoeal disease: a review of active surveillance data. Bulletin of the World Health Organization. 1982; 60(4):605

3. GBD 2013 Mortality and Causes of Death Collaborators. Global, regional, and national age-sex specific all-cause and cause-specific mortality for 240 causes of death, 1990-2013: a systematic analysis for the Global Burden of Disease Study 2013. Lancet, 2015 Jan 10; 385(9963):117-71. (doi: 10.1016/S0140-6736(14)61682-2. Epub 2014 Dec 18).

4. Kotloff KL, Nataro JP, Blackwelder WC, Nasrin D, Farag TH, Panchalingam S, Wu Y, Sow SO, Sur D, Breiman RF, Faruque AS, Zaidi AK, Saha D, Alonso PL, Tamboura B, Sanogo D, Onwuchekwa U, Manna B, Ramamurthy T, Kanungo S, Ochieng JB, Omore R, Oundo JO, Hossain A, Das SK, Ahmed S, Qureshi S, Quadri F, Adegbola RA, Antonio M, Hossain MJ, Akinsola A, Mandomando I, Nhampossa T, Acácio S, Biswas K, O'Reilly CE, Mintz ED, Berkeley LY, Muhsen K, Sommerfelt H, Robins-Browne RM, Levine MM. Burden and aetiology of diarrhoeal disease in infants and young children in developing countries (the Global Enteric Multicenter Study, GEMS): a prospective, case-control study. Lancet, 2013 Jul 20; 382(9888):209-22. (doi: 10.1016/S0140-6736(13)60844-2. Epub 2013 May 14..-13).

5. Rahman AE, Moinuddin M, Molla M, Worku A, Hurt L, Kirkwood B, Mohan SB, Mazumder S, Bhutta Z, Raza F, Mrema S, Masanja H, Kadobera D, Waiswa P, Bahl R, Zangenberg M, Muhe L; Persistent Diarrhoea Research Group. Childhood diarrhoeal deaths in seven low- and middle-income countries. Bull of the World Health Organization. 2014 Sep 1; 92(9):664-71. (doi: 0.2471/BLT.13.134809. Epub 2014 Jun 23).

6. WHO. The treatment of diarrhoea: A manual for physicians and senior health workers. WHO – Geneva 2005

7. WHO. Guidelines for the treatment of Shigellosis. WHO – Geneva 2005

8. Bardhan PK, Beltinger J, Beltinger RW, Hossain A, Mahalanabis D, Gyr K. Screening of patients with acute infectious diarrhoea: evaluation of clinical features, faecal microscopy, and faecal occult blood testing. Scandivian Journal of Gastroenterology. 2000 Jan; 35(1):54-60.

9. Rogawski ET, Meshnick SR, Becker-Dreps S, Adair LS, Sandler RS, Sarkar R, Kattula D, Ward HD, Kang G, Westreich DJ. Reduction in diarrhoeal rates through interventions that prevent unnecessary antibiotic exposure early in life in an observational birth cohort. Journal of Epidemiology and Community Health. 2015 Nov 30. pii: jech-2015-206635. doi: 10.1136/jech-2015-206635

10. Gouws E, Bryce J, Habicht JP, Amaral J, Pariyo G, Schellenberg JA, Fontaine O. Improving antimicrobial use among health workers in first-level facilities: results from the multi-country evaluation of the Integrated Management of Childhood Illness strategy. Bulletin of the World Health Organization. 2004 Jul; 82(7):509-15.

11. Qazi SA, Rehman GN, Khan MA. Standard management of acute respiratory infections in a children's hospital in Pakistan: impact on antibiotic use and case fatality. Bulletin of the World Health Organization. 1996; 74(5):501-7.

12. Sánchez R1, Fernández-Baca V, Díaz MD, Muñoz P, Rodríguez-Créixems M, Bouza E. Evolution of susceptibilities of Campylobacter spp. to quinolones and macrolides. Antimicrobial Agents and Chemotherapy. 1994 Sep; 38(9):1879-82.

13. Treadway G. and Pontani D. Paediatric safety of azithromycin: worldwide experience. Journal of Antimicrobial Chemotherapy. 1996; 37, Suppl. C, 143-149

1. OBJECTIVES

1.1 Primary Objectives:

1. To compare rates of all-cause mortality in the 180 days following enrolment for an episode of high risk diarrhoea without dysentery among children 2 to 23 months of age living in low resource settings who are randomized to receive a 3-day course of azithromycin or placebo.

2. To compare the change in linear growth (ΔLAZ) in the 90 days following enrolment for an episode of high risk diarrhoea without dysentery among children 2 to 23 months of age living in low resource settings who are randomized to receive a 3-day course of azithromycin or placebo.

1.2 Secondary Objectives:

1. To compare changes in markers of acute malnutrition (ΔMUAC and ΔWLZ and Δweight) in the 90 days following an episode of high risk diarrhoea without dysentery among children 2 to 23 months of age living in low resource settings who are randomized to receive a 3-day course of azithromycin or placebo.

2. To evaluate (as determined by verbal and social autopsy- VASA) cause-specific mortality rates across randomization arms in the 180 days following an episode of high risk diarrhoea without dysentery among children 2 to 23 months of age living in low resource settings who are randomized to receive a 3-day course of azithromycin or placebo.

3. To compare the proportion of children hospitalized in the 90 days following enrolment for an episode of high risk diarrhoea without dysentery among children 2 to 23 months of age living in low resource settings who are randomized to receive a 3-day course of azithromycin or placebo.

4. To compare the proportion of children hospitalized or died in the 90 days following enrolment for an episode of high risk diarrhoea without dysentery among children 2 to 23 months of age living in low resource settings who are randomized to receive a 3-day course of azithromycin or placebo.

5. To compare the proportion of children hospitalized or died in the 10 days following enrolment for an episode of high risk diarrhoea without dysentery among children 2 to 23 months of age living in low resource settings who are randomized to receive a 3-day course of azithromycin or placebo.

6. To compare the proportion of strains of *Escherichia coli (E. coli),* isolated from stools, resistant to selected antibiotics at enrolment among the study population. Since children entering later into the trial are coming from the same communities, they can serve as a comparator group indicative of the community level of resistance.

7. To compare the proportion of strains of *E. coli*, isolated from stools, and *Streptococcus pneumoniae* (*S. pneumoniae*), isolated from nasopharyngeal swabs, resistant to selected antibiotics at DAY 90 and DAY 180 in a randomly selected sub-sample of children enrolled in the study and their siblings or close household contacts (children under five years of age living in the same household under the care of the same primary caregiver).

2. SIGNIFICANCE AND IMPLICATIONS

Among specifically targeted children at high-risk of diarrhoea-associated mortality we propose to determine the efficacy of an antibiotic (azithromycin), compared to placebo, in reducing risk of death and linear growth faltering in the six months following an episode of severe diarrhoea. Secondarily, we will evaluate the risk of individual and community-level antibiotic resistance in bacterial enteric and nasopharyngeal infections. These aims will inform future diarrhoea case

management guidelines by empirically determining the potential benefits and harms associated with targeted antibiotic use in children with severe diarrhoea.

2.1 Study sites

The study will be conducted in health facilities in 7 countries in South Asia (Bangladesh, India and Pakistan) and Sub-Saharan Africa (Kenya, Malawi, Mali and Tanzania). The countries have been selected based on study site characteristics as well as the strengths and experience of the country team in conducting large intervention trials. Within each country, 2-10 individual health facilities will be the sites of patient enrolment.

2.2 Study Design

We propose a randomized, multi-country, multi-site, double-blind, placebo-controlled clinical trial. A total of 11,500 children (5,750 per treatment arm) will be enrolled in the study across the 7 countries and followed up for 180 days (Figure 1).

Figure 1. Trial design

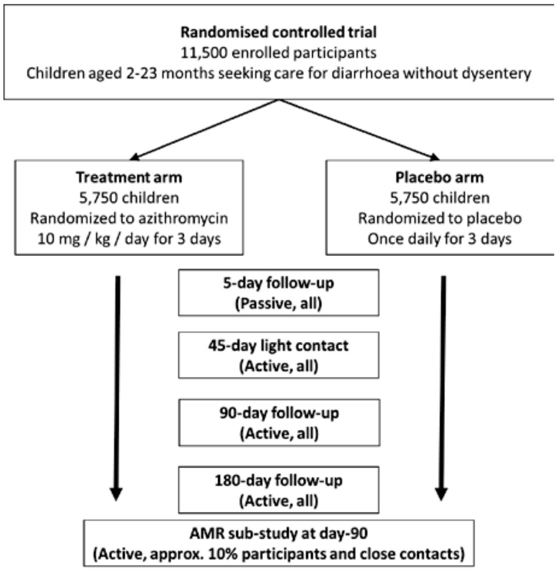

2.3 Eligibility criteria

2.3.1 Inclusion criteria (all of):

- Children aged 2 – 23 months, presenting to a designated health care facility at a participating study site **WITH**
- Diarrhoea per caregiver perception **AND** at least 3 loose or watery stools in the previous 24 hours,
- Diarrhoea for less than 14 days prior to screening **AND** with at least one of the following criteria at presentation:
  A. Signs of some or severe dehydration as per WHO Pocket Book 2013

B. Moderately wasted as defined by a mid-upper arm circumference (MUAC) less than 125 mm (but greater than or equal to 115 mm) or a weight-for-length z-score (WLZ) greater than-3SD and less than or equal to -2SD after rehydration during stabilization period or
C. Severely stunted (length-for-age z-score (LAZ) <-3 SD) **AND**

- Parent or guardian (caregiver) willing to allow household visits on DAY 2 and DAY 3 and willing to return to facility on DAY 90 **AND**
- Parent or guardian (caregiver) provides a consent for trial participation on behalf of the child, based on local standards

Exclusion criteria (any one of):

- Dysentery (gross blood in stool reported by caregiver or observed by healthcare worker [HCW]),
- Suspected *Vibrio cholerae* infection (determined according to WHO guidelines or clinical suspicion),
- Previously or currently enrolled in the ABCD study,
- Concurrently enrolled in another interventional clinical trial,
- Sibling or other child in the household enrolled in the ABCD study and currently taking study medication,
- Signs of associated infections (pneumonia, severe febrile illness, meningitis, mastoiditis or acute ear infection) requiring antibiotic treatment,
- Documented antibiotic use in the 14 days prior to screening (not including standard use of prophylactic antibiotics, i.e. co-trimoxazole use in HIV-exposed children),
- Documented use of metronidazole within the last 14-days,
- Known allergy or contraindication to azithromycin antibiotics,
- Severe acute malnutrition (SAM) defined as WLZ less than −3 SD, or MUAC less than 115 mm, or edema of both feet, OR
- Living too far from the enrolment health center to ensure adequate Directly Observed Therapy (DOT) on DAY 2 and DAY3

2.4 Intervention

3-day course of azithromycin or placebo

2.5 Duration of follow-up

180 days for the main study. For subgroup of 1600- 1800 participants, there will also be a day 180 follow-up visit to collect biological specimens.

3. RATIONALE FOR THE DEFINITION OF STUDY POPULATION AND ELIGIBILITY CRITERIA

3.1 Study Population

This age group (2-23 months) corresponds to a standard age-group defined in IMNCI and used globally to define treatment recommendations. In addition, diarrhoea associated mortality has been demonstrated to be highest in children less than 2 years of age especially in malnourished children.

3.2 Inclusion criteria

Children aged 2-23 months presenting to health facilities for diarrhoea will be screened for enrolment. For this trial, we are seeking to enroll young children at high risk of mortality in the 180 days following an acute diarrhoea episode. This includes children with relatively severe diarrhoea. Since there is no standard definition used in clinical practice for assessment of severity of all diarrhoea in these settings, we propose using signs and symptoms that are usually agreed upon as criteria and objective signs associated with increased risk of death and are also used and understood by health care workers. These signs include those of "some" or "severe" dehydration as per the 2013 WHO Pocket Book for Hospital Care for Children which are established to select the tertile with most severe diarrhoea. In addition, children with evidence of malnutrition (severe stunting or moderate wasting) are at high risk of diarrhoea associated death (associated with as many as 11% of deaths).

3.3 Exclusion criteria

Per current WHO guidelines, children with dysentery, suspected cholera, severe acute malnutrition (SAM), and/or other signs of infections requiring antibiotics should receive antibiotic therapy and therefore, could not be ethically randomized to the placebo arm. As a result, children presenting with a history of bloody stool (per caregiver report or HCW observation), children in a location with a declared cholera outbreak and presenting with presumed cholera, children with SAM, and/or children with signs of associated infections, will be excluded. Associated infections will be suspected if a child has signs of pneumonia/severe pneumonia (cough or difficulty breathing with any one of the following signs: general danger signs as per WHO guidelines, chest in-drawing, or fast breathing), severe febrile illness (or temperature $\geq$ 38.0°C with either danger signs or stiff neck), mastoiditis or acute ear infection (ear problem with any of the following signs: tender swelling behind the ear, pus draining from the ear and discharge reported for less than 14 days, or ear pain).

4. RECRUITMENT

Participants will be identified and recruited by trained study staff from outpatient and inpatient departments of study health facilities. Site specific recruitment plans are detailed in each site implementation strategy document (see attached).

4.1 Screening

After a potentially eligible child has been identified, the study staff will screen the child for eligibility based on the above-mentioned inclusion and exclusion criteria using a standardized screening form (see Core Variable Table). If the child presents with diarrhoea and “no signs” of dehydration, screening will be continued immediately (anthropometric measurements, physical examination, etc.) and the child enrolled if eligible.

4.2 Stabilization period

If the child has signs of “some” or “severe” dehydration, or if the child requires urgent care or the screening physician is unsure of the diagnosis, the child will be kept under observation. During this “stabilization” period, oral and/or IV rehydration will be conducted, and treatment of all urgent conditions will be performed using standard treatment in accordance with the WHO Pocket Book of Hospital Care for Children, 2013. If rehydration of the child is successfully completed, as well as urgent care provided and diagnosis confirmed, screening will be continued (anthropometric measurements, physical examination, etc.) and the child enrolled if eligible. However, if the child is not stabilized or requires additional treatment, the child will not be screened further. The child will be eligible again if stabilized at a later stage, but in this case the screening process will be started all over again.

4.3 Consent

After the child has been found to be eligible to be enrolled in the study, the accompanying primary caregiver will undergo informed consent in his/her local language. During consent, purpose of the study as well as all study procedures will be explained to the caregiver including the administration of the first dose of the study drug by study staff, follow-up visits by study staff to give the second and third doses, and collection of stool and nasopharyngeal samples (see attached Generic Informed Consent Form).

4.4 Enrolment

After the caregiver has agreed to let the child participate in the study and signed the Informed Consent Form, the child will be enrolled and given an enrolment number that will determine the study treatment that this child will receive.

We expect to enroll approximately 105 children per week from all sites combined to reach a target enrolment of 11,500 children within the study period. Individual sites will be allowed to enroll up to 2000 study subjects until the total sample size is achieved.

5. STANDARD MANAGEMENT AND INTERVENTION

Both groups will receive standard of care for diarrhoeal disease, including zinc, rehydration, and nutritional counselling following WHO guidelines. Children with some or severe dehydration will be rehydrated and stabilized prior to completion of screening.

As part of standard case management, caregivers will be advised to seek care immediately if their child is unable to drink or to breastfeed, develops fever, starts passing blood in the stools with continued diarrhoea or becomes sicker. In addition, as per standard WHO guidelines, the caregiver will also be advised to bring their child back to the health facility for a follow up at DAY 5 if the child's clinical status is not improving.

Caregivers will be counseled at enrolment that children with continuing diarrhoea at DAY 5 should return to an appropriate level health facility for further treatment.

5.1 Study Drugs Intervention

Based on the enrolment number, a packet with the same number label containing the intervention bottles for the child will be opened. The packet will contain a study medication displaying child's enrolment number and a specific measuring/dispensing device. These containers will be such that they cannot be contaminated with any external substance and yet be baby friendly to feed the dose. The exact nature of these containers will be finalized by review of options and in consultation with the manufacturer of the drugs. The details will be described in the manual of Standard Operating Procedures (SOP).

The study medication bottle will contain either a 3-day course of azithromycin or placebo and will be given as 1 dose per day (10 mg/kg). The first dose of the study drugs will be given at the health facility by a trained study worker. At this time, the caregiver will be trained on how to administer the study drug at home. On DAY 2 and DAY 3, a study health worker will visit the home of all enrolled children, to provide the subsequent doses of the study drugs or to observe the caregiver giving it.

A fecal sample will be collected and stored from all participants at the enrolment visit, to study the emergence of bacterial resistance to antimicrobial agents among study participants, their household contacts and the community, as described in more detail in section 6.5 below.

5.2 Randomization and blinding

Randomization (1:1) will be done in random sized blocks (with varying block sizes of 4, 6 and 8). All drug and placebo will be procured by WHO MCA Department where a separate randomization list for each of the sites will be generated. This list will then be converted into a unique sequential serial number for each enrolled child for each site. The list provided to the site will only have these serial numbers with the first letter depicting the site. Individual child's supply of study drugs will be provided to each site and labeled with this serial number. At the sites, enrolled children will receive the drugs contained in the bottle with the randomization number identical to the enrolment number. The sites will not have the randomization code.

To ensure blinding, the number of bottles and doses for each of the two groups will be identical. The drug and control bottles and medications will be similar in all aspects including the content, color and taste. Treatment allocation (once assigned) will remain blinded to the participant, the site PI, the study site staff and the hospital clinicians during all data collection phases of the study.

5.3 Study drugs administration

5.3.1 First dose of the study medications

At enrolment the physician/nurse/study worker enrolling the child will administer the first dose of the medication assigned to the child according to his enrolment number. In addition to administering the medication s/he will educate and demonstrate to the caregiver the method of administration as well as the quantity to be administered using the measuring system. This training will help the caregiver to administer doses 2 and 3 which will be given at the child's home (by or under direct observation of the study staff).) In addition, s/he will provide to the caregiver user-friendly instructions in the local language and pictorially, both for quantity and for administration of the assigned drug to be taken at home. In case the child is admitted in the facility all the doses of medication will be administered by the study staff as long the child is admitted. When the child is sent home the education previously imparted will be reinforced.

If a child is enrolled in the afternoon, the caregiver will be instructed to give the second daily dose in the morning of the following day. After administration of the first dose of the medication, the child will be observed for 30 minutes. If vomiting occurs during these 30 minutes, a repeat dose of the medication will be administered. If this dose also is vomited, a second repeat dose will not be given.

The caregiver will be asked to return to the health facility if any of the following signs appear in the next 10 days:

- Anaphylactic reaction, Quincke oedema (angioedema), convulsions, cutaneous rash, arthralgia, urticaria, and severe colitis. (see section on Serious Adverse Events)

5.3.2 Other doses of the study drugs

When the child is sent home (day 1 for outpatient or short admissions and day 2/3 if the child stays in hospital longer) a study worker will accompany the caregiver for ascertainment of the address and location of the house. H/she will deliver the medication bottle with the measuring device to the caregiver at the household and reinforce the message concerning the administration of the subsequent doses.

On Day 2 and Day 3, a study worker will visit the household and observe the caregiver giving the medications to the child in his/her presence or to give the study drugs if the guardian prefers that. In addition, s/he will inquire about the child's symptoms and care after administration of the previous dose. If the child vomits the medication within 30 minutes of the administration of the study medication, a repeat dose will be given and child will be observed for another 30 minutes. A third dose will not be given even if the child vomits again.

On the last dosing day, the study worker will collect the bottle for the completed medication.

6. FOLLOW UP

The follow-up is summarized in Figure 2 below and described in more detail in the subsequent sections.

Figure 2: Follow-up plan

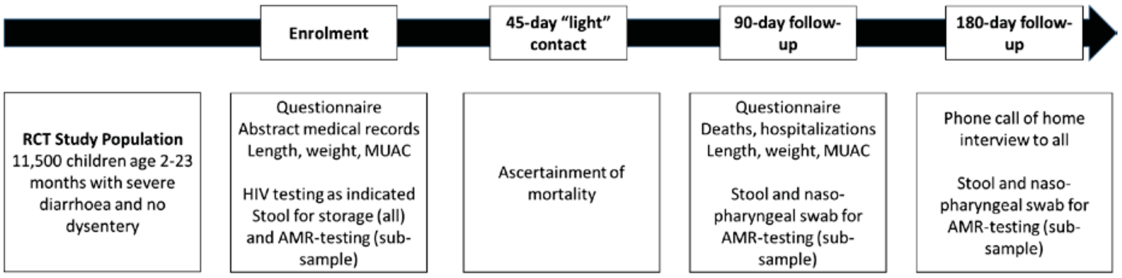

6.1 Day 45 Follow up

Non-medically trained study personnel will contact study participants, using a method of "light contact" at DAY 45 (range DAY 40 to DAY 50). This will involve contact by phone or in-person contact. The purpose of this contact is to ascertain the vital status of the enrolled child at DAY 45, ascertain any hospital admissions since enrolment, and to remind the caregiver of the DAY 90 follow up. No other outcome determination will be performed at this contact.

6.2 Day 90 Follow up

At the DAY 90 follow up a standardized assessment will be performed by an appropriate trained staff to ascertain vital status, hospitalizations, and health of the enrolled child, as well as his/her nutritional status (weight, length and MUAC). Participants will be contacted by phone up to three times within the DAY 80 to DAY 100 period to fix an appointment for this DAY 90 follow up visit that will either take place at the health facility (in which case transport will be reimbursed to the caregiver), or at the home of the enrolled child. Anthropometric measurements will be performed by a team of dedicated personnel, trained according to standard procedures (see SOP manual).

Children enrolled in the AMR sub-study will also be asked to provide a stool sample. In addition, a naso-pharyngeal swab will be taken in the same sub-sample of children. These samples will be processed and used to study the emergence of bacterial resistance to antimicrobial agents, as described in more detail in section 6.5 below.

If a child is reported to have died at any time or at the DAY 45 contact or at the DAY 90 visit, or at any other point during the study follow up period, specially trained study staff will review hospital record (if available) and conduct a standardized verbal autopsy interview to ascertain

the date and cause of death. The verbal autopsy effort will include capturing all relevant hospital/facility-based information on a child that dies in a facility.

6.3 Day 180 Follow up

Study personnel will contact study participants by phone or in person on DAY 180. The purpose of this contact is to ascertain the vital status of the enrolled child at DAY 180 and to ascertain any hospital admissions since day 90 visit. No other outcome determination will be performed at this contact.

An additional follow up contact / visit will be performed for the randomly selected sub-sample of enrolled surviving children who have provided a stool and nasopharyngeal swab sample at the DAY 180 visit and their household contact/sibling. At this visit, a stool sample and a nasopharyngeal swab will be collected and processed as described in detail in section 6.5.

6.4 Outcome measurements

- **Mortality** and date of death to be ascertained at the DAY 45 contact, the DAY 90 and DAY 180 visits, or at any other time point during the 180-day study period. **Verbal and Social Autopsies** (VASA) will be attempted for all recorded deaths within the study period. As part of the VASA autopsy procedures for deaths that occur in facilities, we will collect all available relevant information (diagnostics, treatment and drugs, etc.) from the medical record file of the study subject.
- **Hospitalizations** (number and categorized causes) between days 0 and 180
- **Anthropometric measurements:** length or height, weight and MUAC will be measured at enrolment and at the DAY 90 visit.
- **Antimicrobial resistance** prevalence of phenotypic resistance to selected antibiotics in isolated *E. coli* (stool sample), and *S. pneumoniae* (naso-pharyngeal swabs) at enrolment, at DAY 90 and at DAY 180 will be measured on a sub-sample. Additionally, stool samples will be stored from all children at enrolment, to facilitate later molecular identification of diarrhea etiologies, gut microbiota, and antimicrobial resistance genes in stool bacteria. For the same purpose, stool and nasopharyngeal swab samples will be stored on DAY 90 and DAY 180 from the sub-sample of children (see sections 6.5 and 6.6 for further details).

6.5 Collection and processing of biological specimens

A fecal sample will be collected from all children at enrolment. This sample will be aliquoted and stored at -80°C without further processing, to facilitate later molecular identification and analysis of enteric pathogens and their antimicrobial resistance genes.

A subset of up to 1600 to 1800 ABCD participants (approx. 15%) will be enrolled in a more detailed AMR sub-study. Random assignment into the AMR sub-study will be determined by the WHO Central Coordinating Office as part of the intervention randomization code, stratified by site and intervention arm. Children enrolled in the AMR sub-study will have a stool sample collected and processed at baseline, and a stool sample and a naso-pharyngeal swab collected

and processed for culture and resistance testing at DAY 90 and at DAY 180 after enrolment. In addition, a stool sample and a naso-pharyngeal swab will also be collected from a sibling or other close household contact (child contact) of the enrolled child (index-child) at DAY 90 and DAY 180 180.

The stool samples are primarily collected from whole stool passed by the study participant into a diaper or a container. If, however, the sample is taken at the study clinic (always on enrolment visit, sometimes on subsequent visits), and the child has not passed stool within 2-4 hours of the start of the clinic visit, and the primary caregiver gives a permission to the procedure, the stool sample may be taken with 2 cotton swabs, quickly inserted into the participant's rectum by a research nurse or physician.

Although it is determined at study enrolment whether the index child will participate in the AMR sub-study, no samples will be collected from the siblings or close household contacts before the 90 and 180-day time points. Eligible contacts are the participant's mother or children aged less than 60 months, who have slept in the same household as the enrolled child for 5 of the last 7-nights, and who share the same primary caregiver as the index child. If there are multiple contacts in the household who meet the eligibility criteria, then the preference for inclusion in the "contact study" will be given to the child closest in age and living in the same quarter as the index child. The contact will be the same child at DAY 90 and 180 so as to maintain the ability to evaluate within-individual change in resistance.

For the sub-set of children and their contacts enrolled in the AMR sub-study, before freezing of the stool sample at -80°C, a small amount of stool will be immediately placed in Cary-Blair transport media and shipped to microbiology facilities (maintaining temperatures between 2-8°C) within 24 hours of collection. All collected nasopharyngeal swabs will be placed in skim milk, tryptone, glucose, and glycerin (STGG) transport medium and transported to microbiology facilities for culture and AMR testing.

Upon receipt at the laboratory, stool samples will be plated on MacConkey agar, incubated at 35°C - 37°C for 24 hours, and examined for colonies consistent with *E. coli*. Similarly, naso-pharyneal swabs will be plated onto selective media and incubated at 35°C - 37°C in 5% CO2 overnight and examined for colonies consistent with *S. pneumoniae*. When *E. coli* colonies are identified in the stool samples or *S. pneumoniae* colonies are identified in the naso-pharyngeal samples, distinct colonies will be aggregated into a single cryo vial, aliquoted, and a part will be stored -80°C for later genotypic characterization. The remaining sample will be used for culture-based AMR testing in laboratories at the participating institutions as described below.

The antibiotic susceptibility of the collected *E. coli* or *S. pneumoniae* isolates will be carried out with an automated, commercial microdilution method, using azithromycin and approximately 20 other commonly used antimicrobial agents. For testing the sensitivity of *E. coli* to azithromycin, we will use thin inert non-porous plastic carrier strips, impregnated with a predefined stable antimicrobial gradient of selected antibiotics (so called E-test, each strip containing one antibiotic). Both tests will provide minimum inhibitory concentrations (MICs) for each tested antibiotic.

6.6 Storage of collected biological specimens

The following biological specimens are planned to be stored at -80°C and used for molecular identification of diarrhea etiologies, microbiota, and antimicrobial resistance genes in bacteria and to assess if any of these characteristics is an effect modifier to the intervention effect.

1. At enrolment: Stool samples from all participants
2. At day 90 and day 180: Stool samples from a sub-group (approx. 15%) of participants and their close household contacts, participating in an antimicrobial resistance (AMR) sub-study.
3. At day 90 and day 180: Naso-pharyngeal swab samples from the same sub-group of participants and their close household contacts, participating in the AMR sub-study.

The possibility of sample collection and storage will be separately asked from the caregivers of study participants during the consent process and the caregivers can separately refuse this aspect of the trial. If caregiver consent is obtained, the samples will be stored in secured freezers at each of the seven participating institutions. The stored samples are planned to be analyzed in batches, within four years after all the sample collection for the trial is completed and with up-to-date methods and at sites that have capacity to do those analyses. Any leftover samples will be destroyed at the end of the above-defined storage period. During the storage, only the local PIs and researchers designated by them will have access to the samples.

The study participants or their caregivers can at any point request the discontinuation of storage and destruction of samples collected from them, by contacting the Principal Investigator at her / his study site. Additionally, the study team will strictly adhere to all local regulation at each study site on details of sample storage and use. For any transfer of samples to other locations during the study period, the ABCD team will seek approval from the local IRBs.

7. SERIOUS ADVERSE EVENTS

Serious Adverse Events (SAE) will be recorded from enrolment to DAY 10 (7 days after the end of study intervention). Data on mortality and hospitalizations will be collected until DAY 180.

At enrolment, caregivers will be told to come back to the health facility if the enrolled child present any of the following signs in the following 10 days:

- Anaphylactic reaction, Quincke oedema, convulsions, cutaneous rash, arthralgia, urticaria, and severe colitis.

On DAY 2 and DAY 3 visit for the DOT, the study worker will record any reported SAE.

In case there is a suspected allergic reaction caused by the study drug or other SAE, the participant will not get any further doses of the study drug. In case of a suspected allergic reaction, the study data manager at WHO will break the randomization code for that participant, to know which product the participant was receiving. The data manager will provide this

information to clinicians taking care of the participant, so that they can take in into account in the medical management and also counsel the participant's caregivers about any need to avoid certain drugs in future.

8. TRAINING

Before the initiation of patient enrolment, a one-week training of trainers (TOT) that will include trainers from each site will be conducted under the supervision of the Maternal, Newborn, Child and Adolescent Health (MCA) Department of the WHO at one of the study sites. This TOT will include standardization of all procedures for screening and enrolment (including assessment of dehydration and physical examination), standard case management, randomization, medication dispensing and administration, follow-up procedures, anthropometry assessment and verbal autopsy interviews.

The trainers from each site will then conduct similar intensive training of the study staff in their own respective site prior to initiation of enrolment. Refresher training sessions will be repeated every two months. Records of these training sessions will be kept for examination during external monitoring. In case internal monitoring of the study shows inconsistencies or deviations in the conduct of the study, training sessions on specific elements of the protocol will be conducted as required.

9. TIMELINE

The total study period is estimated at 43 months, from January 1st 2017 to July 31st, 2020. This includes approximately 4 months of preparatory time 30 months of active enrolment (starting on May 1st, 2017), six months of follow-up after last enrolment, and 3 months for data cleaning, analysis and dissemination.

10. DATA ANALYSIS

An outline of the analysis plan for the primary objectives is presented here.

Primary analyses will be intent-to-treat (ITT) based on randomization allocation to the 3-day course of azithromycin or placebo. For each analysis, azithromycin will be compared to those treated with placebo.

The primary end-points in this trial are proportion of deaths in the 180 days post-enrolment and change in length-for-age z-score in the 90 days post-enrolment by study arm

Deaths will be ascertained by study staff either through family reports, community health workers, hospital staff, or determined at follow-up visits. Date will be determined by verbal autopsy or medical records in cases of facility deaths.

Length will be assessed using a length board at enrolment and at the day 90 follow-up visit by study staff trained in anthropometry. Length will be measured twice at each visit, a third time if the two repeated measures varied by more than 10%, and the median length used for analysis. Length-for-age z-scores will be calculated using the WHO ANTHRO software. Implausible length values will be considered on a case-by-case basis within each site and implausible lengths set to missing.

Loss to follow-up will be determined at 180 days after enrolment and defined as non-attendance at the 180-day follow-up visit and inability to be contacted after three subsequent attempts. All analyses will utilize a complete case analysis other than in time-to-event analyses where children who are lost to follow-up will be censored at the date of last contact with the study team. All hypothesis testing will utilize an alpha of 0.05 to determine statistical significance.

10.1 Primary Objectives

1. *To compare rates of all-cause mortality in the 180 days following enrolment for an episode of high risk diarrhoea without dysentery among children 2 to 23 months of age living in low resource settings who are randomized to receive a 3-day course of azithromycin or placebo.*

Period prevalence of death will be compared between randomly-assigned treatment groups using relative risk regression. Mortality will be defined as a binary variable and will be defined as any event of death from time of randomization to end of day 180. Time to death will not be considered. Censored individuals will be retained in the denominator. For the primary ITT analysis, the following model will be used:

$$\boldsymbol{E(Y|randomisation\ arm) = e^{(\beta_0+\beta_1 X_{azm})}}$$

where x_{azm} is an indicator variable specifying randomized to the azithromycin group (x_{azm}=1) or not (x_{azm} =0). The risk ratio comparing the risk of death in children randomized to azithromycin vs. placebo will be determined by e^{β}. The statistical significance of this comparison will be determined by Wald test.

If the baseline assessment of randomization reveals an imbalance in characteristics between the randomization arms, we will evaluate these characteristics as potential confounders in a sub-analysis secondary to the ITT. The following baseline characteristics will be evaluated for balance between randomization arms (median age, stunting prevalence, wasting prevalence, prevalence of “some” and “severe” dehydration, site [Bangladesh, India, Kenya, Malawi, Mali, Pakistan, Tanzania], hospital admission, and median duration of diarrhoea). Potential baseline confounders will be added stepwise in the relative risk model with randomization arm as follows:

$$\boldsymbol{e^{(\beta_0+\beta_1 X_{azm}+\beta_i X_i)}}$$

Where *i* are the baseline characteristic(s) included in the model. These will be maintained in the model if their inclusion in the model changes the hazard ratio by more than 10%. Also, in a sub-

analysis, separate analysis for each site (Bangladesh, India Kenya, Malawi, Mali, Pakistan, Tanzania) will be presented to describe differences in treatment effects between sites (no formal hypothesis tests within each site stratum will be performed).

In a per-protocol analysis, also secondary to the ITT analysis, relative risk regression will be fit as described above among the subset of children with documented completion of the full course of treatment (direct observation of 3 daily doses and) with at-risk-time beginning on date of observed third dose.

2. *To compare changes in linear growth (ΔLAZ) in the 90 days following enrolment for an episode of high risk diarrhoea without dysentery among children 2 to 23 months of age living in low resource settings who are randomized to receive a 3-day course of azithromycin or placebo.*

Analysis strategy: Linear regression model will be used to compare mean ΔLAZ across the treatment groups in surviving children. ΔLAZ will be operationalized as the difference in LAZ between the 90-day follow-up visit and LAZ at baseline. For the primary ITT analysis, the following model will be used:

$$E(Y|randomisation\ arm) = \beta_0 + \beta X_{azm}$$

where Y=mean ΔLAZ, and x_{azm} is an indicator variable of being randomized to the azithromycin group (x_{azm}=1) or not (x_{azm}=0). The mean difference in ΔLAZ in children randomized to azithromycin vs. placebo will be determined by (β). The statistical significance of this comparison will be determined by independent t-test.

If the baseline assessment of randomization reveals an imbalance in characteristics between the randomization arms, we will evaluate these characteristics as potential confounders in a sub-analysis secondary to the ITT as described in the mortality time to event analyses. Potential baseline confounders will be added stepwise in the linear regression model with randomization arms and maintained in the model if inclusion in the model changes the effect estimates (difference in means) by more than 10%.

In a per-protocol analysis, also secondary to the ITT analysis, a linear regression model will be fit as described above among the subset of children with documented completion of the full course of treatment (direct observation of 3 daily doses).

10.2 Secondary Objectives

1. *To compare changes in markers of acute malnutrition (ΔMUAC and ΔWLZ and Δweight) in the 90 days following an episode of high risk diarrhoea without dysentery among children 2 to 23 months of age living in low resource settings who are randomized to receive a 3-day course of azithromycin or placebo.*

Linear regression (and associated z-test) will be used to compare mean ΔMUAC and ΔWLZ and weight across the treatment groups among surviving children.

2. *To evaluate (as determined by Verbal and Social Autopsy – VASA) cause-specific mortality rates across randomization arms in in the 180 days following an episode of high risk diarrhoea without dysentery among children 2 to 23 months of age living in low resource settings who are randomized to receive a 3-day course of azithromycin or placebo.*

We will conduct Cox regression and K-M survival analyses (as described for aim 1) for time to cause-specific mortality (diarrhoea, pneumonia, malnutrition, other) as separate endpoints to understand intervention effects on specific causes of death.

3. *To compare the proportion of children hospitalized in the 90 days following enrolment for an episode of high risk diarrhoea without dysentery among children 2 to 23 months of age living in low resource settings who are randomized to receive a 3-day course of azithromycin or placebo.*

4. *To compare the proportion of children hospitalized or dying in the 90 days following enrolment for an episode of high risk diarrhoea without dysentery among children 2 to 23 months of age living in low resource settings who are randomized to receive a 3-day course of azithromycin or placebo.*

5. *To compare the proportion of children hospitalized or dying in the initial 10 days following enrolment for an episode of high risk diarrhoea without dysentery among children 2 to 23 months of age living in low resource settings who are randomized to receive a 3-day course of azithromycin or placebo.*

6. *To compare the presence of strains of E. coli resistant to selected antibiotics at enrolment between children in the different treatment arms entering the trial over time. Since children entering later into the trial are coming from the same communities, they can serve as a comparator group indicative of the community level of resistance.*

Prevalence of resistance to selected antibiotics and (with Wilson binomial 95% confidence intervals) will be calculated on all samples collected in Year 1 of the study, in Year 2 of the study and Year 3 of the study and compared using a chi-square test to determine the secular trend of evolution of antibiotic resistance at each site.

7. *To compare the presence of strains of E. coli, isolated from stools, and S. pneumococcus, isolated from nasopharyngeal swab, resistant to macrolide, β-lactamase and quinolone at DAY 90 and DAY 180 in a randomly selected sub-sample of children enrolled in the study and other children under age 5 years living in the same household.*

Prevalence of antibiotic resistance as a function of treatment group and time will be modelled using generalized estimating equations (GEE), with a Poisson link and exchangeable correlation structure for each antibiotic and each isolate, to account for repeated measurements within individuals. The GEE model will include three time-points (baseline, DAY 90 and DAY 180) for antibiotic resistance data from *E. coli* isolates and two time-points (DAY 90 and DAY 180 for *S. pneumoniae* isolates). Pairwise comparisons by treatment groups (azithromycin vs. placebo) at DAY 90 and DAY 180 and between baseline and DAY 180 (among the *E. coli* isolates) will be conducted using Wald tests. A Wald test will also be used to test the hypothesis

that the magnitude of change in prevalence, between treatment groups, differs at 90 days and 180 days (effect modification by time). Because multiple hypothesis tests will be performed (4 Wald tests per each of 8 antibiotics per each of 2 isolates types =56 tests) we will report p-values adjusted for multiple comparisons using a Bonferroni correction or a similar method.

For the siblings and close contacts, the same analytical methods will be used. However, no *E. coli* data from baseline will be collected therefore the *E. coli* GEE antibiotic resistance models will contain only two time points (DAY 90 and DAY 180).

10.3 Sample size

For the purposes of sample size determination for the ABCD trial, we have estimated that the 180-day mortality in the control group will be 2.7%. Assuming this baseline mortality, a relative risk in the intervention group of 0.65 (35% reduction in mortality in the intervention group), 90% power, 95% confidence, assumed loss to follow-up 10%, and 1:1 ratio in the numbers of participants in the control and intervention group, the required sample size would be 5,696 per group or 11,392 in total. Since there is an initial plan to conduct one interim analysis for safety (under Fleming bounds, assuming equally spaced analyses and maintaining total alpha=0.05 and beta=0.1, and two-sided symmetric bounds), the sample size will be inflated by a factor of 1.009[1]. Hence, the total planned sample size will be 5,750 per group and 11,500 in total.

The estimated overall mortality in the control group is a summary function of earlier data from the GEMS study as well as the assumed proportions of children with various risk factors in the sample and respective sub-group specific mortality rates. The 60-day mortality rate in children under five years of age with moderate to severe diarrhoea or dysentery in the GEMS study averaged 2.0% across all sites. Excluding the two low mortality sites in Bangladesh and West Bengal (India), this mortality rate was 2.8%. The mortality estimates for children with various degrees of stunting or wasting come from GEMS 1a study (which covers children with less severe diarrhoea), supported by meta-analyses of some 10 child cohorts, collected between 1977 and 1997 (Black et at, Lancet Nutrition Series 2008; Olofin et al PLoS One, 2013).

As the study is implemented in 7 countries, on average each country will need to enroll 1645 children (about 822 per study group). Over an enrolment period of 30 months, this would mean approximately 13 children/country will need to be enrolled per week. Individual sites will be allowed to enroll up to 2000 study subjects until the total sample size is achieved.

Aim 2 will compare change in length-for-age Z-score (ΔLAZ) between the control group and intervention from enrolment to 90 days. We have estimated that we will have 80% power to detect at least a 0.04 difference in mean ΔLAZ between study groups using a two-sided t-test to compare the difference in mean change in LAZ between two groups with $\alpha=0.05$ and standard deviation (SD) of 0.7 in both groups. This is comparable to results observed in the Global Enterics Multicenter Study (GEMS). As the SD of the difference in ΔLAZ has been

[1] Jennison C, Turnbull BW. Group Sequential Methods with Applications to Clinical Trials. Chapman and Hall/CRC,1999 (page 150)

shown to vary, Table 1 below outlines the minimum detectable difference in ΔLAZ with varying SD.

Table 1. Sample size and minimum detectable difference in ΔLAZ with varying power and standard deviation

Scenario	Minimum detectable difference in mean Δ*LAZ*	Standard deviation (equal SD per group)	Sample size (per group) at 90% power	Sample size (per group) at 80% power
A	0.06	0.7	2862	2138
		0.6	2103	1571
		0.5	1461	1092
B	0.05	0.7	4120	3078
		0.6	3028	2262
		0.5	2103	1571
C	0.04	0.7	6437	4809
		0.6	4730	3533
		0.5	3285	2454

We have estimated that we will have 90% power to detect at least a 0.04 difference in mean ΔLAZ between study groups (5,750 children per group) using a two-sided t-test to compare the difference in mean ΔLAZ between two groups with α=0.05 and standard deviation (SD) of 0.6 in both groups. Assuming 50% of children will be less than 12 months of age at enrolment (2,85085 infants per group), we will have 90% power to detect at least a 0.06 difference in mean ΔLAZ between study groups comprised of infants and separately, between study groups comprised of toddlers (12-23 months). As a point of reference, combined across all sites in GEMS, Moderate to Severe Diarrhoea (MSD) cases lost 0.04 more in LAZ, on average, than controls among infants ($p<0.001$) and among toddlers, MSD cases lost 0.10 more in LAZ than controls ($p<0.001$).

For secondary objectives 6-7, the number of children enrolled in the AMR sub-study was determined for comparison of azithromycin resistance prevalence between the azithromycin and placebo arm on DAY 90 and DAY 180. The analysis is planned to be done regionally (separately for Asia and Africa), because we assume similarity in AMR within a region but differences between the two regions. Hence, all the numbers below indicate numbers / region.

The minimum isolate number required per treatment group was calculated assuming an alpha of 0.05 for two-way antibiotic resistance prevalence comparisons between randomization arms. We assumed a 15-20% prevalence of azithromycin resistance in the placebo arm at DAY 90, a slightly more conservative estimate than azithromycin-resistance estimates among *Shigella* isolates in Mirpur Bangladesh (22%) and in *E.coli* isolates in the Kongwa District of Tanzania (19%). We assumed that the increase in AMR would be about 2-fold at 90 days post enrolment. This is based on studies comparing azithromycin resistance in *S. pneumoniae* and E. coli between communities with and without community-wide azithromycin distribution which found resistance prevalence in the azithromycin-treated communities to be at least two-times

higher at 3 (30% vs. 10%) and 6 (23% vs. 12%) months after the antibiotic administration compared to untreated communities.

To achieve 90% power to detect a two-times higher prevalence in the azithromycin-treated arms, compared to 20% prevalence in the comparison group, we will need 119 *E. coli* and 119 *S. pneumoniae* isolates per treatment arm. We set this as the minimum number of children and siblings or close household contacts, from whom specific bacterial colonies would be available at the last data collection point on day 180. Accounting for losses at various points of study (as indicated in Table 2 below), a total of 345 children per arm (or 690 in total) would need to be recruited from both study regions (Asia and Africa). Given all the uncertainties in our assumptions and for logistical ease, we agreed to collect up from 1600 to 1800 study subjects allowing each site to collect isolates from approximately 250 children to this sub-study. For the four sites in Africa, this translates to approximately 1000 children enrolled in the AMR study and approximately 750 for the three sites in Asia

Table 2. Assumed losses of participants at the various stages of the AMR sub-study

Point in study	Cause of loss	% loss	Number of children / arm
Randomization to the AMR substudy (day 0)	Done at enrolment	0%	345
Available at day 90 visit	Death, other loss to follow-up between days 0 and 90	10%	311
1st sampling of study child (day 90)	Refusal of AMR sampling	20%	248
Recruitment of sibling or household contact (day 90)	No sibling or household contact available	20%	199
Identification of specific bacteria (day 90)	No colonization with the specific bacteria	33%	133
2nd sampling of participant & sibling (day 180)	Death, other loss to follow-up	10%	119

The death or loss to follow-up estimate (10%) is the same as used for the main sample size estimation. Refusal of AMR sampling (20%) and non-availability of any sibling (20%) are the research groups predictions on the average situation at the various sites and the bacterial colonization rates are based on earlier data that *S. pneumoniae* carriage proportions range from 65-81% and *E.coli* grows in approximately 76-91% of stool cultures.

11. RISK MITIGATION

Azithromycin is approved for infants older than 6 months of age and has been widely used without significant safety concerns in neonates and infants for a wide-range of bacterial infections. Safety and pharmacokinetics have been evaluated in newborn infants with gestational ages as low as 24 weeks (13). While several case reports of pyloric stenosis have

been reported in infants receiving azithromycin, the FDA OND Maternal Health Team concluded in 2010 that available data do not demonstrate an association between azithromycin use and increased risk of pyloric stenosis[2]. However, to monitor possible incidence of this adverse reaction, all study participants will be queried about hospitalizations (for abdominal surgery, for diarrhoea and for some other defined causes) at the day 45 and day 90 follow-up visits.

Large-scale use of antibiotics raises concern for the possibility that antimicrobial resistance will be induced and ultimately could negatively impact clinical outcomes in the communities in which resistance emerges. Several studies have examined the emergence of antibiotic-resistance among pharyngeal pneumococcal isolates following mass treatment with azithromycin (9). Multiple studies have documented relatively rapid emergence of macrolide resistance following mass drug administration with azithromycin (9) although rates of resistance decline quickly after discontinuation of treatment (12). To monitor possible incidence, spread, and disappearance of antimicrobial resistance, a subgroup of participants and their siblings or close household contacts will be asked to provide stool and naso-pharyngeal swab specimens before, during, and after the trial intervention, as described in more detail in section 6.5.

Current efforts to reduce antimicrobial use are failing and new strategies and approaches to change prescribing practices and patient demand factors are urgently required. Given the widespread and inappropriate use of antibiotics, efforts to identify and focus use in a sub-population of children with well-defined and highly specific acute diarrhoea episodes could reduce overall use. This dynamic has been observed for other infectious diseases[3][4]. The ABCD Trial results, if robust, might thus, paradoxically, lead to reduced use of antibiotics and lessen the ecologic pressure pushing resistance. To further limit antibiotic use, the ABCD trial enrolment criteria have been made as strict as possible, only including children whom a guardian brings to a health facility and who has at least moderate dehydration, moderate wasting or severe stunting.

The mortality estimates used to determine the sample size required to achieve adequate power and based on a large multi-center observational study (GEMS), a related observational study (GEMS1a) and meta-analyses of earlier cohort studies in Sub-Saharan Africa and Southern Asia. Although the estimates used are considered conservative based on other available data and preference was given to the selection of sites thought to experience high diarrhoea associated mortality, it is conceivable that there will be fewer mortality events observed in this trial, which would have implications for study power.

---

[2] Eberly MD, Eide MB, Thompson JL, Nylund CM Azithromycin in Early Infancy and Pyloric Stenosis. American Academy of Pediatrics. Published Online (date) February 16, 2015, doi: 10.1542/peds.2014-2026

[3] Qazi, S.A, Rehman, G.H, Khan, M.A. Standard management of acute respiratory infections in a children's hospital in Pakistan: impact on antibiotic use and case fatality. Bull. WHO, 1996, 74 (5): 501-507

[4] Gouws, E. Bryce, J., Habicht, J.P., et. al. Improving antimicrobial use among health workers in first-level facilities: results from the Multi-Country Evaluation of the Integrated Management of Childhood Illness strategy. Bull. WHO, 2004 82 (7): 509-515

12 STUDY MANAGEMENT AND OVERSIGHT

12.1 Coordination

The WHO coordinators in the MCA will be responsible for the oversight of the trial, including harmonization of methods and procedures across the study sites. The MCA, WHO, is responsible for developing technical guidelines, including the management of infections in under-five-year-old children. MCA has the operational advantages of building on existing WHO facilities at the International, Regional and Country level. This includes a communications network and a managerial group with experience in successful handling of projects of a similar size and complexity. MCA also has a large network of collaborators in academic and other institutions around the world, which have different expertise in epidemiology, clinical, microbiological, radiological and diagnostic areas of childhood illnesses including diarrhoeal disease. The WHO coordinating team will provide technical support in the development of site protocols, site-specific study instruments and standard operating procedures. The WHO coordinating team will also oversee randomization and will be also responsible for procuring the antibiotic and placebo, arranging for their adequate packaging into full-treatment courses and delivering the packaged treatments to the study sites.

The WHO coordinating team will develop guidelines for monitoring and evaluation of the progress of the trial and will be responsible for internal and external monitoring and oversight of quality assurance procedures. WHO study coordinators and others identified by them will ensure that at least two structured monitoring visits are conducted to each site every year. The monitoring visits will have as their primary aim quality control and the improvement of study implementation. The monitors will make direct observations of all relevant study procedures and data management activities. The content of monitoring will vary in response to the stage of study implementation. Monitoring will start with a review of the sites' readiness to begin implementation. After implementation has started, it will shift its focus to the adequacy of procedures for recruitment (including informed consent and adherence to enrolment criteria), treatment delivery and follow-up. More intensive monitoring visits are planned during the first six months of study implementation than in the later part of the study. This process is intended to ensure early problem-identification and prompt resolution at the individual site level and the identification of any variability of procedures across sites that might require rectification.

12.2 Local Governance

A Principal Investigator (PI) from each site will be the leader of the local research team and will be responsible for the execution of the agreed protocol and for the integrity of the data. The PI will be responsible for local financial management as well as for obtaining the necessary local approvals for conducting such a study.

12.3 Ethical Review

The study protocol and associated documents (consent forms, CRFs, etc.) will be submitted to the Ethical Review Committee (ERC) at the WHO and the Institutional Review Boards at each participating institution for ethical approval.

12.4 Trial Advisory Group and Technical Steering Committee

The Trial Advisory Group (TAG) will provide advice to the WHO coordinating team. This small group of leading experts will provide strategic guidance and assist with independent advice in the case of difficult issues in the conduct of the trial. The Technical Steering Committee (TSC) will be comprised of the co-PIs from each study site and include members from the WHO coordinating team and the Bill & Melinda Gates Foundation. The initial tasks for TSC will include assisting in the finalization of the site-specific protocols and study instruments. The TAG and TSC will review the progress in the implementation of the study, the planning and review of the data analyses and presentation of the study results. WHO will facilitate meetings of the TAG at least once per year, whereas the TSG will meet at least monthly, normally electronically.

12.5 Data Safety and Monitoring Board

A Data Safety and Monitoring Board (DSMB) has been established by WHO at study initiation to monitor severe adverse events (SAEs) and to approve the statistical analysis plan and associated stopping rules for benefit, futility, or harm determined using O'Brien-Fleming stopping boundaries. This initial meeting will be held before patient accruals are initiated. The DSMB includes five members with expertise in clinical trials, statistics, child mortality assessment, ethics, and pediatric care in resource limited settings and it is represented by both study regions. The DSMB will meet electronically or in person at least every 6 months and an in person meeting of the DSMB will occur at least one time per year. Severe adverse events (SAEs) related to study participation will be monitored in real-time and will be summarized and reported to study investigators, WHO and relevant institutional review boards (IRBs) within 48 hours of occurrence. On a monthly basis, frequencies and descriptions of SAEs will be pooled by the data management team at WHO and circulated to investigators, DSMB and IRBs. When approximately half of the person-time is accrued in the study, the DSMB will review an interim data analysis by arm to determine whether stopping boundaries have been crossed.

12.6 Quality assurance

Field supervisors will be responsible for assuring the training of the field staff is of high quality and rigorous. They will schedule the testing and retraining as required at their individual site. Assessment of individual study personnel's abilities to use the standardized enrolment criteria and conduct the anthropometric measurements consistently across the study population are key responsibilities. They also will be accountable for the site-specific approaches to minimizing loss-to-follow-up. These quality assurance approaches will be reinforced by the WHO coordinating team during the early site visits.

Field supervisors will check all forms completed by field workers before data entry. Data cleaning quality assurance will be performed using consistency and range checks both at the study sites and at the WHO data coordination Centre as described in the data management section. Data quality checks will also be applied on a quarterly basis at the WHO and feedback will be provided to the Principal Investigators and study managers in all sites.

PIs will receive brief monthly progress reports from the Data Coordinating Center during the entire study period and will participate in regular telephone conferences with WHO staff. The monthly progress reports will include the number children assessed, number of children recruited, home visits due to be conducted, actual visits conducted, child hospitalizations, deaths and VASA conducted. The templates of the monthly progress report will be developed by WHO Data Coordination Centre with inputs from the sites.

The trial will be registered by the WHO as a clinical trial in one or more Primary Registries in the WHO Registry Network.

12.7 Data management

The WHO coordinating team, working in collaboration with RTI, will ensure harmonization of data collection and data management processes across all sites. All sites will collect information on a core set of variables with standard definitions. The WHO will provide a set of range and consistency checks that must be applied to these variables, although the exact procedures used to carry out these checks will be left to the individual sites. As a general principle, there should be maximum flexibility for each of the sites since they have been selected on the basis of their capabilities to carry out large trials.

Each site will be responsible for data entry and initial cleaning of the data, including running range and consistency checks as well as periodic reviews of distributions and identification of outliers. Each study site will resolve any inconsistencies within their database in consultation with their field data collection team and with field verification if needed. Individual sites will be required to provide data on the core set of variables in SQL readable format monthly to a central data repository established for the project at the WHO. The WHO team will run another set of range and consistency checks including checking of consistency of data quality across sites quarterly. Any inconsistencies or queries will be notified to the study site, which will be expected to check and address the list of queries and resubmit data. Cleaned data from all sites will be pooled and stored in a SQL database at the WHO. Data analysis workshops will be held in Geneva following completion and unblinding of the trial data.

The WHO Coordination group will maintain close communication with both the PI`s and the Bill & Melinda Gates Foundation assigned Program Officer. Monthly check in calls to review progress and challenges will take place between the WHO and BMGF. In addition, BMGF will be invited to attend all meetings of the Trial Advisory Group.

12.8 References

1. Liu L, Johnson HL, Cousens S, et al. Global, regional, and national causes of child mortality: an updated systematic analysis for 2010 with time trends since 2000. Lancet, 2012; **379**:2151-2161.

2. Snyder JD, Merson MH. The magnitude of the global problem of acute diarrhoeal disease: a review of active surveillance data. Bulletin of the World Health Organization. 1982; **60**(4):605

3. GBD 2013 Mortality and Causes of Death Collaborators. Global, regional, and national age-sex specific all-cause and cause-specific mortality for 240 causes of death, 1990-2013: a systematic analysis for the Global Burden of Disease Study 2013. Lancet, 2015 Jan 10; **385**(9963):117-71. (doi: 10.1016/S0140-6736(14)61682-2. Epub 2014 Dec 18).

4. Kotloff KL, Nataro JP, Blackwelder WC, Nasrin D, Farag TH, Panchalingam S, Wu Y, Sow SO, Sur D, Breiman RF, Faruque AS, Zaidi AK, Saha D, Alonso PL, Tamboura B, Sanogo D, Onwuchekwa U, Manna B, Ramamurthy T, Kanungo S, Ochieng JB, Omore R, Oundo JO, Hossain A, Das SK, Ahmed S, Qureshi S, Quadri F, Adegbola RA, Antonio M, Hossain MJ, Akinsola A, Mandomando I, Nhampossa T, Acácio S, Biswas K, O'Reilly CE, Mintz ED, Berkeley LY, Muhsen K, Sommerfelt H, Robins-Browne RM, Levine MM. Burden and aetiology of diarrhoeal disease in infants and young children in developing countries (the Global Enteric Multicenter Study, GEMS): a prospective, case-control study. Lancet, 2013 Jul 20; **382**(9888):209-22. (doi: 10.1016/S0140-6736(13)60844-2. Epub 2013 May 14..-13).

5. Rahman AE, Moinuddin M, Molla M, Worku A, Hurt L, Kirkwood B, Mohan SB, Mazumder S, Bhutta Z, Raza F, Mrema S, Masanja H, Kadobera D, Waiswa P, Bahl R, Zangenberg M, Muhe L; Persistent Diarrhoea Research Group. Childhood diarrhoeal deaths in seven low- and middle-income countries. Bull of the World Health Organization. 2014 Sep 1; **92**(9):664-71. (doi: 0.2471/BLT.13.134809. Epub 2014 Jun 23).

6. WHO. The treatment of diarrhoea: A manual for physicians and senior health workers. WHO – Geneva 2005

7. WHO. Guidelines for the treatment of Shigellosis. WHO – Geneva 2005

8. Bardhan PK1, Beltinger J, Beltinger RW, Hossain A, Mahalanabis D, Gyr K. Screening of patients with acute infectious diarrhoea: evaluation of clinical features, faecal microscopy, and faecal occult blood testing. Scandivian Journal of Gastroenterology. 2000 Jan; **35**(1):54-60.

9. Rogawski ET, Meshnick SR, Becker-Dreps S, Adair LS, Sandler RS, Sarkar R, Kattula D, Ward HD, Kang G, Westreich DJ. Reduction in diarrhoeal rates through interventions that prevent unnecessary antibiotic exposure early in life in an observational birth cohort.

Journal of Epidemiology and Community Health. 2015 Nov 30. pii: jech-2015-206635. doi: 10.1136/jech-2015-206635

10. Gouws E, Bryce J, Habicht JP, Amaral J, Pariyo G, Schellenberg JA, Fontaine O. Improving antimicrobial use among health workers in first-level facilities: results from the multi-country evaluation of the Integrated Management of Childhood Illness strategy. Bulletin of the World Health Organization. 2004 Jul; **82**(7):509-15.

11. Qazi SA, Rehman GN, Khan MA. Standard management of acute respiratory infections in a children's hospital in Pakistan: impact on antibiotic use and case fatality. Bulletin of the World Health Organization. 1996; **74**(5):501-7.

12. Sánchez R1, Fernández-Baca V, Díaz MD, Muñoz P, Rodríguez-Créixems M, Bouza E. Evolution of susceptibilities of Campylobacter spp. to quinolones and macrolides. Antimicrobial Agents and Chemotherapy. 1994 Sep; **38**(9):1879-82.

13. Sudha Chaudhari, Pradeep Suryawanshi, Shrikant Ambardekar, Manoj Chinchwadkar and Arun Kinare. Safety Profile of Ciprofloxacin used for Neonatal Septicemia. Indian Pediatrics, 2004; **41**:1250.

14. Jennifer A. Goldman, Gregory L. Kearns. Fluoroquinolone Use in Paediatrics: Focus on Safety and Place in Therapy. 18th Expert Committee on the Selection and Use of Essential Medicines (2011).

15. Treadway G. and Pontani D. Paediatric safety of azithromycin: worldwide experience. Journal of Antimicrobial Chemotherapy. 1996; **37**, Suppl. C, 143-149

13 SITE IMPLEMENTATION PLANS

13.1 Bangladesh

<u>Study Area</u>

The study will take place in two hospitals (Dhaka hospital and Mirpur hospital) of the International Centre for Diarrhoeal Disease Research, Bangladesh (icddr,b), situated in Dhaka city in Bangladesh. The two hospitals partly cater to the needs of Dhaka city having a population of more than 13 million. The Dhaka Hospital of icddr,b is the largest diarrhoeal disease hospital in the world. We expect mortality rates up to day 90 of follow up in both the hospitals to be higher at around 2%. In Bangladesh, diarrhoea was responsible for almost 20% of total deaths in 1980 but 6% of 0.119 million under-five deaths in 2015 [2], even following the treatment and prevention of dehydration with oral rehydration salts (ORS) and/or intravenous (IV) fluids, administration of zinc, feeding advice, and with antibiotic treatment for cases of bloody diarrhoea and cholera [3]. Dhaka Hospital is a 350 bed hospital, provides care and free treatment to around 140,000 diarrhoeal patients with or without complications each year. On an average 250 patients are treated in the hospital each day. However, during the epidemic season (observed at least twice a year), the hospital has to treat as many as 1200 patients per day. More

than 80% of the patients come from poor socio-economic backgrounds from urban and peri-urban Dhaka. About 42% of the patients fall within the age group of 2-23 months. Among the children aged 2-23 months, 35% present with moderate to severe diarrhoea (MSD) and 29% with mid-upper arm circumference (MUAC) >115 to <125 mm. The Mirpur hospital of icddr,b is a 100 bed hospital treating diarrhoeal patents for the last 5 years and data on proportion of 2-23 months children, MSD, and MUAC are almost similar as in the Dhaka hospital. Enteric pathogens potentially treatable with an antibiotic are common (ETEC [8%], Shigella [6%], and Cholera [5%]) at these two urban hospitals [4]. Among Shigella isolates, ciprofloxacin azithromycin sensitivity ranges from 60-76% to. However, sensitivity of both the drugs in cholera and salmonella was around 100%.

Recruitment

Recruitment will begin at the Dhaka and Mirpur hospitals after IRB approval (both WHO and icddr,b). A subject enrolment and follow up team (includes physicians, nurses, research assistants, health workers, and heath attendants) responsible for enrolled patients at day 1 and during subsequent hospitalization (if required) and another mobile team (includes research assistants, health workers) focused solely on community sensitization, directly observed therapy, and follow-up into the community will be placed at each of the two hospitals. Study staff will work closely with hospital staff to determine when there is a potentially eligible child either in the oral rehydration therapy (ORT) corner or in the short stay ward (SSW). The children with some or severe dehydration will be treated in the SSW until dehydration is fully corrected. Stabilization/rehydration period will not exceed 6 hours before enrolment. The ORT corner manages diarrhoeal children without dehydration and those do not require any antibiotics and are observed for 2 hours. Children with no dehydration but having moderate malnutrition using MUAC (>115 to <125 mm) or having moderate stunting (<-2SD for length for age of the WHO median) or moderate wasting (< = 2SD for weight for length of the WHO median) will be enrolled from ORT. Children with MSD will be enrolled from the SSW. The study co-coordinator (senior medical officer) and study physician will also participate in the daily morning health discussion to sensitize caregivers about the study and actively identify potentially eligible participants. The follow-up team will be deployed into the community as per the protocol and they will decide whether a child needs to seek care at a facility as per standard WHO guideline.

Screening/Enrolment

After an eligible child has been identified with the screening described above, the study research assistant will approach the primary caregiver of the child and describe the study procedures. The research assistant will explain that the child and caregiver must be willing to be visited at their home for directly observed therapy (DOT) for the next two days, be willing to be visited at their home for follow-up 90-days after enrolment, and be willing to provide a stool sample from the child at enrolment and at the 90 and 180 day follow ups (for a subset of children). If the caregiver is interested in participating, the study staff will obtain informed consent. Informed consent will be conducted in local language (Bangla). The parent or guardian (primary caregiver) must sign written informed consent (or provide a witnessed thumbprint if not literate) prior to enrolment.

The study physician will interview the primary caregiver of the child to collect required information in the case report form. A stool sample will be collected and processed according to study protocol. The follow-up research assistant will collect detailed contact information from the caregivers using a standardized patient locator form, including drawing of a map of home location with the help of the caregiver. We will also ascertain mobile phone information of the caregiver for adherence assessment, follow-up visit reminders, and home tracing. If a caregiver does not have a personal mobile phone we will obtain the mobile phone information of a household member or friend of the caregivers' choosing. We expect less than 5% of participants to be without cell phone access based on our recently accomplished post discharge follow-up study in severely malnourished pneumonia children [6].

Following consent, each participant will be assigned a unique study Patient Identification Number (PID). A card detailing the PID and the medical personnel responsible for enrolment will be given to the primary caregiver of enrolled children. The patient locator form will be the only link between the PID and the participant's name and will be stored in a locked file cabinet at each site accessible by only the study physician/nurses and follow-up staff for the purpose of patient tracing. Patient enrolment in Dhaka hospital will be done 24 hours a day, 7 days a week and in Mirpur hospital from 9 am to 5 pm in each day.

Standard of Care

All enrolled children will receive standard of care for diarrhoea management per WHO and Bangladesh National Health guidelines.

In children with acute diarrhoea, this will include:

a. Standardized assessment of hydration status

b. Rehydration and maintenance fluids with intravenous or oral rehydration solutions (ORS) as indicated

c. Oral zinc therapy for 10-14 days (10 mg for infants < 6 months old; 20 mg for 6 months and older)

d. Recommendation for increased fluid intake and continued feeding at home

e. Instructions on when to seek follow-up care.

Intervention

Following consent, appropriate acute management, and stool sample collection, the study physician will identify the pre-labeled intervention bottle corresponding to the PID. The study physician will explain the regimen and the importance of adhering to the intervention. Direct observation of first dose of study medication will be performed by study staff at the hospitals. In addition, direct observation of the doses on day 2 and day 3 will be conducted by the mobile team in the household on day two and day three. At the end of each day at the enrolment centers, the assigned health worker of the mobile study team will work with the respective assigned research assistant to determine the visitation schedule for subjects enrolled earlier that day and walk through the patient locator form.

Follow-up

Between 40 and 50 days since enrolment, a follow-up will be conducted to establish contact and ascertain vital status. The preferred method of contact for the 45-day follow-up will be mobile phone. If no telephone number is provided, or if the participant cannot be reached after four attempts, the mobile study team will conduct a home visit to ascertain vital status and remind the caregiver of the 90-day follow-up visit. However, if any child is found to have any illness during the contact, the child will be encouraged to visit relevant health facilities.

All enrolled children will be scheduled to be visited at their home for follow-up at 90 days (between day 80 and day 100) following enrolment to ascertain vital status and anthropometric measurements. The research assistant will give a phone call reminder one day prior to the scheduled visit. During this visit, anthropometric measurements will be obtained by the study personnel. Transportation cost will be reimbursed at the follow-up visit thereby incentivizing participants to return. If the participant does not return at their scheduled time, study staff will attempt to make contact with the primary caregiver via cell phone; if no telephone number is provided, or if the participant cannot be reached, study staff will trace the child to the household and bring the caregiver and child to the enrolment center if at any follow-up, a caregiver reports that a child has died, study personnel trained in verbal autopsy will review hospital records (if available) and will conduct a standardized verbal autopsy at the household to ascertain the potential cause of death.

Data Management

All data will be collected in real-time using an offline computer-automated questionnaire installed on tabs at the sites at each study day. The database is password-protected and all site staff responsible for entering data will have unique usernames and passwords that are controlled by the data manager/study statistician in each study sites and will only have rights to enter data. The data manager/study statistician evaluates data daily and generates reports on a weekly basis to identify any missing or erroneous data. On a monthly basis, the central data manager (WHO based) will enable cross-checking of data entered by generating a detailed monthly report. Changes to existing data will be tracked using an electronic auditing system and adverse events will be recorded in the database.

Ethical Assurance for Protection of Human rights

The study protocol and data collection tools, and the informed consent forms to be used in the study will be submitted to the Research Review Committee (RRC) and Ethical Review Committee (ERC) of icddr,b, and the study would be initiated only after receiving approval of the committee. All medical information, description of treatment, and results of the laboratory tests performed will be kept under lock and key, only research staff, hospital physicians and nurses, and ERC/DSMB members of icddr,b will have access to these information.

References

1. National Institute of Population Research and Training (2011) Bangladesh Demographic and Health Survey 2011.

2. UNICEF (2015) Committing to Child Survival: A Promise Renewed. Progress Report 2015.

3. WHO (2005) The treatment of diarrhoea: a manual for physicians and other senior health workers, 4th rev. Geneva; WHO.

4. Das SK, Ahmed S, Ferdous F, Farzana FD, Chisti MJ, et al. (2013) Etiological diversity of diarrhoeal disease in Bangladesh. Journal of infection in developing countries 7: 900-909.

5. World Health Organization. Chart Booklet: Integrated Management of Childhood Illness. Geneva, Switzerland. WHO 2014.

6. Chisti MJ, Graham SM, Duke T, Ahmed T, Faruque AS, et al. (2014) Post-discharge mortality in children with severe malnutrition and pneumonia in Bangladesh. PLoS One 9: e107663.

13.2 India

Description of the Study Site

The study will be undertaken in Meerut district of Western Uttar Pradesh, India covering a population of 3,720,000 (~14.6% constitutes the under-five population). District is divided into 3 Tehsils: Mawana, Meerut and Sardhana and 12 blocks: Kharkhoda, Daurala. Hastinapur, Jani, Machhara, Mawana Kala,Meerut, Pariskhitgarh, Rajpura, Rohata, Sarurpur and Sardhana. Both public (Department of Health and Family Welfare) and private sector agencies (hospitals, nursing homes and clinics) provide health services in Meerut district. The site carries a high disease burden in terms of water borne diseases like diarrhoea, dysentery and typhoid, acute respiratory illness especially in young children. As per the Annual Health survey of Meerut (2011-2012), the incidence of diarrhoea is about 11.7% in children 0-6 yrs (Rural 12.8% and urban 9.9%) (upnrhm.gov.in/site-files/IMR_AHS.pdf; Data from the CMO Office, Meerut). Overall mortality associated with diarrhoeal diseases is estimated to be 0.13%. The urban poor constitute a high-risk group with regard to child health and have a substantially higher prevalence of child morbidity and mortality due to malnutrition and infectious diseases. A study conducted in the slums of Meerut reported the prevalence of diarrhoea to be as high as 24.2% among the under five children (UHRC, 2007-08).

Study Recruitment Facilities and Catchment Area

The proposed study will be carried out in two tertiary care hospitals [1 private- Subharti Medical College (SMC) and 1 government- Lala Lajpat Rai Memorial (LLRM) Medical College], 1 district hospital and 9 of the 12 CHCs in rural areas and their catchment area. Diarrhoea caseload in these facilities is high as most of the children (meeting criteria for "moderate to severe diarrhoea") get referred to these facilities. As part of the National Rural Health Mission (NRHM) ASHA workers have been providing community health services. They will be used for referring diarrhoeal cases to the facilities.

Catchment Area: In rural setting we have identified the villages covering ~1 lakh population to define the catchment area under each CHC. In urban setting, the city area around the health facilities (~7-10 Kms radius) has been identified as catchment area.

Community mobilization: Before the start of the study discussions will be held with the Director, NRHM and CMO, Meerut district. PI and Co-PI will visit all the selected health facilities and explain the study purpose to the health personnel. Village level meetings will be held to mobilize the community support and generate awareness.

Study Team: The study team will have four different levels of study personnel with specific roles and responsibilities. The study team will include Project manager, one study coordinator, one-supervisor/quality control personnel at SMC, one at LLRM, 1 at District Hospital and one at each of the CHCs. They will work closely with the hospital staff to identify and recruit children. Additionally mobile study team – 2 members at SMC, 2 at LLRM, 2 at district hospital, 3 at CHCs (covering 3 CHCs per person) will be deployed. These members will be provided motorbikes and they will be responsible for direct observational therapy and follow up visits. In addition there will be Lab manager, data manager, lab staff, and anthropometry and quality control team members.

Screening and Enrolment

A surveillance system will be set up at SMC, LLRM and District hospital OPD/Emergency wards will identify all diarrhoea cases and screen them for eligibility according to study procedures. For those children meeting the eligibility criteria, enrolment supervisor will approach the parents/caregiver and explain the study procedures to them. H/She will explain that the child and caregiver must be willing to be visited at home (or at a pre-determined nearby facility) for directly observed therapy (DOT) for the next two days, be willing to return to the health facility for the follow-up visit on day-90 after enrolment, and be willing to provide a stool sample from the child at enrolment and possibly at 90 days (for a subset of randomly selected children). If the caregiver is interested in study participation, informed written consent will be obtained in local language (Hindi).

After obtaining consent, the enrolment supervisor will complete the Enrolment form and record the baseline information such as socio-demographic characteristics, breastfeeding and vaccination history. A stool sample will be collected in a sterile stool container and processed according to study protocol

Following consent, each participant will be assigned a unique subject identification number. A card will be given to the caregiver of enrolled children providing contact information in case of an emergency. Subject identification information will be stored in a locked file cabinet accessible by only the project manager and study coordinator for the purpose of patient tracing.

At the time of discharge contact details including phone number of the caregiver or relative/friend (in case personal number not available) will be obtained to send follow-up visit reminders and for follow up visits We will also take the help of ASHAs (part of government health system) working in the community to locate the houses.

Standard of Care

All enrolled children will receive standard of care for diarrhoea management per WHO and National Health guidelines.

In children with acute diarrhoea, this will include:

a. Standardized assessment of hydration status
b. Rehydration and maintenance fluids with intravenous or oral rehydration solutions (ORS) as indicated
c. Oral zinc therapy for 10-14 days (10 mg for infants < 6 months old; 20 mg for 6 months and older)
d. Recommendation for increased fluid intake and continued feeding at home
e. Instructions on when to seek follow-up care.

Delivery of Intervention

Following enrolment, stool sample collection, and acute management, the study supervisor will identify the pre-labeled intervention bottle corresponding to the Subject ID. The supervisor will explain the treatment regime and visitation schedule of the mobile team for monitoring the intervention dose in the subsequent 2 days and will counsel the caregiver on the importance of adhering to the intervention. Study supervisor will monitor the first dose administered to the child at the health facility. Enrolled subjects information will be entered into the database in real time and follow-up visit schedules will be prepared every evening for visits falling on the next day. The program manager along with study supervisor will allocate visits to mobile team workers of a particular area. In case the child is still in hospital information will be sent to the hospital supervisor. All children enrolled will be visited on Day-2 and 3 by the mobile team to observe the dose of intervention given. Outcome information will be recorded in a predesigned questionnaire in the netbook. Subjects needing medical attention will be advised to visit health facilities. For children still in hospital, hospital supervisor will give the dose.

Follow-up Visits

A follow-up contact will be established between day 40 and 50 to ascertain vital status (usually a phone call). If in case the contact couldn't be established with the family after four attempts the study worker will be advised to visit the household. A standardized questionnaire will be used to record the vital status of the child and to remind the caregiver of the day-90 follow-up visit at the facility.

All enrolled children and caregivers will be scheduled to visit the health facility on day-90 (between day-80 and -100) following enrolment to ascertain vital status and record anthropometric measurements. Prior to the scheduled visit i.e. 2 days in advance text message reminders will be sent to the mobile phone number provided. During this visit, the study supervisors stationed at the facility will take anthropometric measurements and mortality information since the last time the child was seen by study staff. If the participant does not return at their scheduled time, study supervisor will attempt to make contact with the caregiver via mobile phone; if the contact could not be established study team member will visit the participant household and facilitate them to visit the nearest health facility.

If at any of the follow-up visits, a caregiver reports that a child has died an appointment for verbal autopsy will be set up at home to ascertain the cause of death. The study staff trained in verbal autopsy will review the hospital records (if available) and conduct interviews in the local language 'Hindi' describing the events that preceded the death.

Quality Control: For overall supervision, two mobile supervisory staff will be recruited to perform quality checks.10% among the enrolled households will be selected randomly from the visit schedule using a computer algorithm. Quality control team will visit these households to check if the required visitation by the mobile community team were conducted as per protocol. In 5% households they will collect repeat data, which will be used by the program manager to verify the accuracy of data. Random quality controls check of anthropometry data and repeat measurement will be done in visits requiring anthropometric measurements.

Data Management: All data will be electronically captured in real time using laptops in the facilities and tablets or netbooks in the community. At the end of each day, all data from various facilities and community will be synchronized with the central server. The database will be password-protected and all site staff responsible for entering data will have unique usernames and passwords controlled by the data manager. The data manager will generate the follow-up visit schedules on daily basis for each facility. Additionally, the data manager will evaluate data daily and generate reports on a weekly basis to identify any missing information or erroneous data. An audit trail will be maintained for recording any changes to the existing data.

Ethical Review:

The study protocol and associated research instruments will be submitted to the Subharti Medical College Ethical Review Board and Lala Lajpat Rai Memorial College Ethical Review Board for approval.

13.3 Kenya

Study Area

The study will take place in Homa Bay and Migori counties in the Nyanza region of Kenya, which covers a population of 1.9 million people living within the two counties (50% of which are under age 15) and a total area of 5780km^2. Cases of moderate-to-severe diarrhoea (MSD) from the GEMS Kenya site in the Demographic Surveillance System (DSS) area of the nearby Kisumu County, had a case-fatality rate of 3.5%.[1] The current study will be conducted outside of the DSS site where we expect mortality rates to be higher, and where we are conducting an ongoing diarrhoea surveillance study. Eight district, 12 sub-district, and four missionary hospitals serve Homa Bay and Migori County and are the referral centers for dispensaries and health centers. Enteric pathogens potentially treatable with an antibiotic are common (ETEC [4.4%], EPEC [6.3%], *Shigella* [5.2%]) at three district hospitals in the region (Homa Bay, Migori, and Kisii).[2] Over 25% of children presenting with acute diarrhoea have at least one bacterial pathogen that may be treatable with an antibiotic (ETEC, EPEC, *Shigella*, *Campylobacter*, and EAEC), an expected prevalence that is likely underestimated due to culture-based methods used for bacteria isolates other than for detection of diarrhoeagenic *Escherichia coli*. Among *Shigella* isolates identified in this study, ciprofloxacin resistance has

not been identified while 90.5% were resistant to trimethoprim/sulfamethoxazole, 47.6% to ampicillin, and 74.6% to tetracycline.[3] The HIV prevalence among children presenting with acute diarrhoea is 5.2% and 11.3% of HIV-uninfected children are HIV-exposed.

The government, through the Ministry of Health, is the main provider of health services in the study area. The various health facilities are graded from Level 1 to 5 depending on the services offered and lower level facilities refer to higher level facilities depending on illness severity. Level 1 facilities offer community-level health education and promotion services provided by community health workers. Level 2 Dispensaries and Level 3 Health Centre's largely provide outpatient services and limited health care and laboratory services. Level 4 sub-County and Level 5 County hospitals offer both inpatient and outpatient services, and limited laboratory and radiological services.

Private and faith-based health facilities complement the services offered by the government clinics at a minimal cost. These facilities generally offer inpatient, outpatient, and laboratory services as well as routine immunizations. These facilities collaborate with the department of public health in disease surveillance and research activities within the study area.

Recruitment

Enrolment will begin at six sub-county hospitals (Awendo, Isebania, Mbita, Ndhiwa, Rongo, and Rachuonyo) in Homa Bay and Migori county. Additional sub-country hospitals within Homa Bay and Migori counties (Migori, Taranganya, Uriri, Ogwedhe, Matosa, Suba, Rangwe, Kendu Bay, and Macalder) may be added if necessary for enrolment numbers. Three permanent study staff (a clinical officer, study nurse, follow-up counsellor) will be placed at each of the selected hospitals. Study staff will work closely with hospital staff to determine when there is a potentially eligible child either on the ward or in the outpatient unit, a system that is currently used to recruit children in our ongoing diarrhoea surveillance study. The study counsellor will also participate in the daily morning health talk to sensitize caregivers about the study and actively identify potentially eligible participants. To further enrich enrolment, a mobile team focused on community sensitization, directly observed therapy (DOT), and participant follow-up will be deployed in the community. Each enrolment centre will be assigned a mobile team member who will travel throughout the community via motorbike.

Screening/Enrolment

After a potentially eligible child has been identified, the study nurse will screen them for eligibility according to study procedures. Study staff will explain that the child and caregiver must be willing to be visited at the home (or at a pre-determined nearby location) for directly observed therapy (DOT) for the next two days, be willing to return to the health facility 90-days after enrolment, and be willing to provide a stool sample from the child at enrolment and possibly at 90 and 180 days (for a subset of randomly selected children). If the caregiver is interested in participating, informed consent will be obtained. Informed consent will be conducted in the language of the respondent's choosing (English, Kiswahili, Kuria, or Luo). The parent or guardian (primary caregiver) must sign written informed consent (or provide a witnessed thumbprint if not literate) prior to enrolment.

If laboratory results (including HIV and malaria status) are not available from the hospitalization record, a finger/heel prick will be used to collect a small amount of blood for malaria thin/thick smear or rapid diagnostic testing, and HIV testing will be performed as indicated by Kenya NASCOP Guidelines (HIV testing should be performed in all children presenting to a healthcare facility). Any child newly diagnosed with HIV will be referred to the HIV Care Clinics at the study sites for follow-up care and treatment. The study staff will interview the primary caregiver of the child to collect information on socio-demographic characteristics, breastfeeding and vaccination history, and the HIV status of the primary caregiver. A stool sample will be collected in a sterile stool container and processed according to the study protocol. The follow-up counsellor will collect detailed contact information from the caregivers using a standardized patient locator form, including drawing of a map of home location by study staff with the help of the caregiver as well as using Google Maps satellite to confirm the drawn map. We will also ascertain mobile phone information of the caregiver for follow-up visit reminders and home tracing. If a caregiver does not have a personal mobile phone we will obtain the mobile phone information of a household member or friend of the caregivers' choosing. We expect less than 5% of participants to be without cell phone access based on our ongoing diarrhoea surveillance study.

Following consent, each participant will be assigned a unique study Patient Identification Number (PID). A card detailing the PID and the medical personnel responsible for enrolment will be given to the primary caregiver of enrolled children. The patient locator form will be the only link between the PID and the participant's name and will be stored in a locked file cabinet at each site accessible by only the study clinician and follow-up staff for the purpose of patient tracing.

<u>Standard of Care</u>

All enrolled children will receive standard of care for diarrhoea management per WHO and Kenya Ministry of Health guidelines.

In children with acute diarrhoea, this will include:

a. Standardized assessment of hydration status

b. Rehydration and maintenance fluids with intravenous or oral rehydration solutions (ORS) as indicated

c. Oral zinc therapy for 10-14 days (10 mg for infants < 6 months old; 20 mg for 6 months and older)

d. Recommendation for increased fluid intake and continued feeding at home

e. Instructions on when to seek follow-up care.

<u>Intervention</u>

Following consent, stool sample collection, and acute management, the study nurse will identify the pre-labelled intervention bottles corresponding to the PID. The study nurse will explain the regimen and plan for direct observation, directly observe administration of the first dose, and

will provide information-motivation-behaviour (IMB) theory based counselling on the importance of adhering to the intervention. Direct observation of first dose of study medication will be performed by study staff at the enrolment site. In addition, direct observation of the doses on DAY 2 and DAY 3 will be conducted by the mobile team in the household on day two and day three. At the end of each day at the enrolment centres, the assigned member of the mobile study team will work with the facility-based study team to determine the visitation schedule for patients enrolled earlier that day and walk through the patient locator form with the counsellor.

Follow-up

Between 40 and 50 days since enrolment, a follow-up will be conducted to establish contact and ascertain vital status of the enrolled child. The preferred method of contact for the 45-day follow-up will be mobile phone. If no telephone number is provided, or if the participant cannot be reached after three attempts, the mobile study team will conduct a home visit. A standardized script and brief questionnaire will be used to ascertain vital status and to remind the caregiver of the DAY 90 follow-up visit.

All enrolled children and primary caregivers will be scheduled to return to the health facility at DAY 90 (between 80 and 100) following enrolment to ascertain vital status and anthropometric measurements. Phone call reminders will be sent to the phone number provided 1 day prior to the scheduled visit. In addition, study staff will call participants to actively remind them of the scheduled visit. During this visit, anthropometric measurements and stool sample will be obtained by the study personnel. Transportation cost will be reimbursed at the follow-up visit thereby incentivizing participants to return. If the participant does not return at their scheduled time, study staff will attempt to make contact with the primary caregiver via cell phone; if no telephone number is provided, or if the participant cannot be reached, study staff will trace the child to the household and bring the caregiver and child to the enrolment centre. If at any follow-up, a caregiver reports that a child has died, study staff trained in verbal autopsy will review hospital records (if available) and will conduct a standardized verbal autopsy at the household to ascertain the cause of death.

Data Management

All data will be collected in real-time using an offline computer-automated questionnaire installed on netbooks at the sites. At the end of each study day, the site staff will sync the offline version of the database with an online secure SQL server. The database is password-protected and all site staff responsible for entering data will have unique usernames and passwords that are controlled by the data manager in Nairobi and will only have rights to enter data. The Nairobi-based data manager evaluates data daily and generates reports on a weekly basis to identify any missing or erroneous data. On a monthly basis, the Seattle-based data manager will cross-check the data being entered by generating a detailed monthly report. Changes to existing data will be tracked using an electronic auditing system and adverse events will be recorded in the database. In case the offline electronic data entry system is not working, paper forms will be used to collect data and entered in the online database when internet is available.

Ethical and Regulatory Considerations

The study will be conducted according to Good Clinical Practice (GCP), the Declaration of Helsinki, Institutional Review Boards and local rules and regulations of Kenya. Submission of the protocol and any protocol amendments to regulatory agencies will occur in accordance with local regulatory requirements. Kenya Medical Research Institute, Scientific and Ethical Review Unit (KEMRI-SERU) is the IRB of record for the Kenyan Site. This protocol will be submitted to the KEMRI-SERU for review and approval. After approval by KEMRI-SERU, it will be submitted to the Expert Committee on Clinical Trials (ECCT) of the Pharmacy and Poisons Board (PPB) at the Kenya Ministry of Health for review and approval before the protocol is implemented.

13.4 Malawi

Study Area

Blantyre district in southern Malawi has a population of 1.3 million, one large government referral hospital (the Queen Elizabeth Central Hospital, QECH) and 23 government primary health centres. These facilities treat between 1000-3000 children with non-bloody diarrhoea each year. Hospitalised non-bloody diarrhoea from the established VacSurv surveillance programme (examining rotavirus vaccine effectiveness) has 6-week case-fatality of 1.7%. Data on overall mortality associated with diarrhoeal diseases is lacking. HIV prevalence among children presenting with acute diarrhoea is 7% and 20% of HIV-uninfected children are HIV-exposed. Stunting occurs in 27% of children with diarrhoea in our surveillance, but in Malawi DHS 2010 stunting prevalence was 49%. Surveillance for rotavirus, norovirus and bacterial pathogens is ongoing. Enteric pathogens potentially treatable with an antibiotic are common. Among rotavirus negative stool specimen presence of bacterial pathogens was common (Campylobacter 18%, Shigella 12%, Salmonella 1.2%, and verotoxin-producing E. coli 1%). A substantial proportion of children presenting with acute diarrhoea have at least one bacterial pathogen that may be treatable with an antibiotic, an expected prevalence that is likely underestimated due to culture-based methods used for bacteria isolates.

Recruitment

Recruitment will occur at QECH and four primary health centres – Bangwe, Ndirande, Limbe and Zingwangwa. Each site will have a team comprising 1 clinical officer, 2 study nurses and 3 field workers. Study staff will screen potentially eligible children attending study facilities for the treatment of non-bloody diarrhoea. A study nurse will also participate in the daily morning health talk to sensitize caregivers about the study and actively identify potentially eligible participants. Children will be triaged and facility care provided as per standard IMCI guideline, including rehydration, zinc provision, nutritional advice. Families will be encouraged to attend for review 5 days later. Children requiring referral will be advised to attend QECH and the study team alerted of their scheduled arrival. Transport costs will be supported for children needing referral.

A field team will conduct directly observed therapy for children enrolled in the study, and perform 90-day follow-up household visits. Anthropometry will be conducted at the 90 day visits and referrals made for under-nutrition if clinically indicated.

Screening/Enrolment

After a potentially eligible child has been identified they will be screened for eligibility according to study protocol. The study nurse will approach the primary caregiver and describe the study procedures and obtain informed consent. Caregiver must be willing to be visited at the home (or at a pre-determined nearby location) for directly observed therapy (DOT) for the next two days, and at 90 day for follow-up visit or be willing to return to the health facility, and must be willing to provide a stool sample from the child at enrolment and at 90 days. If the caregiver is interested in participating, study staff will obtain informed consent. Informed consent will be conducted in Chichewa in the first instance, or English if preferred by the family. The parent or guardian (primary caregiver) must sign written informed consent (or provide a witnessed thumbprint if not literate) prior to enrolment. Emergency treatment will be provided prior to any consent procedures for children with severe illness or IMCI danger signs and consent procedures commenced only after stabilization.

Rapid diagnostic test for malaria and HIV testing will occur in adherence with national guidelines. Any child newly diagnosed with HIV will be referred to the HIV Care Clinics at the study sites for follow-up care and treatment. After the clinical examination and while ongoing rehydration and care is continuing under review of the study clinician, the study nurse will interview the primary caregiver of the child to collect information on socio-demographic characteristics, breastfeeding and vaccination history. A stool sample will be collected in a sterile stool container and processed according to study protocol.

Study staff will collect detailed contact information from the caregivers using a standardized patient locator form, including drawing of a map of home location by study staff with the help of the caregiver as well as confirm the drawn map by geocoding using Blantyre's ePal technology. Mobile phone contact information of the caregiver will be recorded for adherence assessment, follow-up visit reminders, and home tracing. If a caregiver does not have a personal mobile phone we will obtain the mobile phone information of a household member or friend of the caregivers' choosing. We expect less than 25% of participants to be without cell phone access based on our ongoing diarrhoea surveillance study.

Following consent, each participant will be assigned a unique study Patient Identification Number (PID). A card detailing the PID and the medical personnel responsible for enrolment will be given to the primary caregiver of enrolled children, and a sticker with the PID placed on the health passport. The patient locator form will be the only link between the PID and the participant's name and will be stored in a locked file cabinet at each site accessible by only the study clinician and follow-up staff for the purpose of patient tracing.

Standard of Care

All enrolled children will receive standard of care for diarrhoea management per WHO and National Health guidelines.

In children with acute diarrhoea, this will include:

a. Standardized assessment of hydration status

b. Rehydration and maintenance fluids with intravenous or oral rehydration solutions (ORS) as indicated

c. Oral zinc therapy for 10-14 days (10 mg for infants < 6 months old; 20 mg for 6 months and older)

d. Recommendation for increased fluid intake and continued feeding at home

e. Instructions on when to seek follow-up care.

Intervention

Following consent and stool sample collection, the study nurse will identify the pre-labeled intervention bottle corresponding to the PID. The study nurse will explain the regimen and plan for direct observation, directly observe administration of the first dose, and will provide information-motivation-behavior (IMB) theory based counseling on the importance of adhering to the intervention. Direct observation of first dose of study medication will be performed by study staff at the enrolment site. In addition, direct observation of thedaily doses on day 2 and day 3 will be conducted by the mobile field team in the household on day two and day three (exact timing will be in coordination with the caregiver by phone if possible). At the end of each day at the enrolment centers, the assigned member of the mobile study team will work with the facility-based study team to determine the visitation schedule for patients enrolled earlier that day and walk through the patient locator form with the counselor.

Follow-up

Between 40 and 50 days following enrolment, contact will be made with the caregiver to ascertain vital status of the participating child, preferably by telephone. If no telephone number is provided, or if the participant cannot be reached after four attempts, the mobile field team will conduct a home visit. A standardized script and brief questionnaire will be used to ascertain vital status and to remind the caregiver of the 90-day follow-up visit.

All enrolled children and primary caregivers will be visited at home or scheduled to return to the health facility at 90 days (between 80 and 100) following enrolment to ascertain vital status and anthropometric measurements and collect stool samples. Text message reminders will be sent to the phone number provided 2 and 1 day prior to the scheduled visit. During this visit, anthropometric measurements will be obtained by the study personnel. Transportation cost will be reimbursed at the follow-up visit. If the participant does not return at their scheduled time, study staff will attempt to make contact with the primary caregiver via cell phone; if no telephone number is provided, or if the participant cannot be reached, study staff will trace the child to the household and bring the caregiver and child to the enrolment center. If at any follow-up, a caregiver reports that a child has died, study staff trained in verbal autopsy will review hospital records (if available) and will conduct a standardized verbal autopsy at the household to ascertain the cause of death.

Data Management

All data will be collected in real-time using offline electronic data capture installed on tablets at the sites. At the end of each study day, the site staff will perform data backup. Data will be

collected from each site and collated weekly to a central secure SQL server. All backup data will be encrypted. The database is password-protected and all site staff responsible for entering data will have unique usernames and passwords that are controlled by the data manager and will only have rights to enter data or make modifications in real time but not to change previously entered and saved data. The data manager evaluates data and generates reports on a weekly basis to identify any missing or erroneous data. On a monthly basis, the study coordinator will check all data and generate a detailed monthly report, an encrypted version of which will be sent electronically to the WHO Geneva. Changes to existing data will be tracked using an electronic auditing system and adverse events will be recorded in the database.

The study protocol and associated research instruments will be submitted to the University of Malawi College of Medicine Research Ethics Committee and Liverpool University Ethical Review Board for review and approval prior to initiating the study. Study drug will be approved by the Malawi Poisons, Medicines and Pharmaceuicals Board.

13.5 Mali

Study area

Mali (**Figure 1**) is a land-locked country in West Africa (population ~15.3 million) ranked by the United Nations in 2014 as the 12th least developed country. Only one third of adults achieve literacy. According to UNICEF (State of the World's Children, 2014), Mali's has the world's 8th highest under 5 mortality rate (128 per 1000 live births), with similarly high mortality rates for neonates (42 per 1000 live births) and infants (80 per 1000 live births). Other national indicators that may have particular relevance for diarrhoeal diseases in children include access to improved drinking water (89% of urban and 53% or rural households) and improved sanitation (25% of urban and 14% of rural households). Use of oral rehydration solution (ORS) for diarrhoea is 11%. Most women (80%) report exclusive breast feeding for at least 6 months. Moderate and severe underweight, stunting, and wasting are present in 19%, 28%, and 9% of children 0-59 months of age, respectively. Nutritional factors are estimated to contribute to 45% of childhood deaths in Mali. HIV seroprevalence remains low (~1.2%).

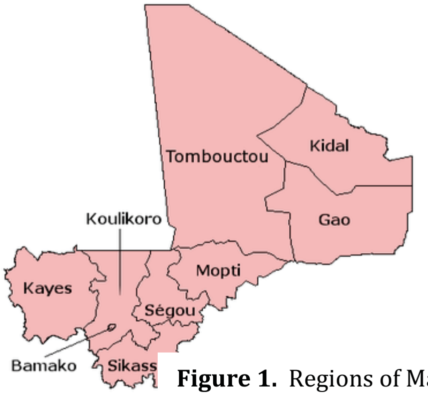

Figure 1. Regions of Mali.

Proposed Study Sites for the Current Antibiotic Treatment for Diarrhoea Study.

Sample size estimates for the current study are based on an expected mortality rate of 3% among the control group. Although it is difficult to predict with precision, data based on GEMS (**Table 1**) and GEMS 1a suggest that mortality among children at the GEMS sites who are eligible for the current study may be lower than 3%. We plan to compensate for this in the selection of sampling frames for the current study.

	Cases		Controls		OR (95% CI)
	Total*	Number of deaths (%)	Total*	Number of deaths (%)	
Manhiça, Mozambique	681	51 (7·5%)	1296	11 (0·85%)	13·4 (6·1–29·3)†
Basse, The Gambia	1029	39 (3·8%)	1569	7 (0·58%)	7·0 (3·0–16·5)†
Nyanza Province, Kenya	1476	52 (3·5%)	1883	11 (0·50%)	5·5 (2·8–10·7)†
Karachi (Bin Qasim Town), Pakistan	1258	16 (1·3%)	1838	1 (0·05%)	13·1 (0·99–172·4)†
Bamako, Mali	2033	23 (1·1%)	2064	5 (0·24%)	5·5 (1·8–16·5)†
Mirzapur, Bangladesh	1394	7 (0·50%)	2465	1 (0·04%)	12·4 (2·0–77·5)‡
Kolkata, India	1568	2 (0·13%)	2014	1 (0·05%)	2·6 (0·34–19·6)‡
All Sites Combined	9439	190 (2·0%)	13 129	37 (0·28%)	8·5 (5·8–12·5)†

Table 1. Mortality among MSD cases and their matched controls at 7 GEMS sites during the 50-90 day follow-up

Figure 2.

Two sampling frames are proposed for enrolment of an estimated 2,150 children: the first includes 2 health facilities in urban Bamako and the second includes 2 health facilities in rural Koulikoro (**Figure 1**). **In Bamako**: we will recruit from l'Hôpital Gabriel Touré (HGT), the major paediatric tertiary care center in Mali, and from the CSREF located in Commune VI (**Figure 2**). At HGT, recruitment will be ongoing for the Vaccine Impact on Diarrhoea in Africa (VIDA) study among children from the two quarters belonging to our DSS. For the current study, we will recruit eligible children who live in the remaining 55 quarters in Bamako. Recruiting from health care facilities in Bamako but outside of our DSS will offer the advantage of accessing a population [illegible]itization, household visits, etc. which could result [illegible]t).

Figure 2.

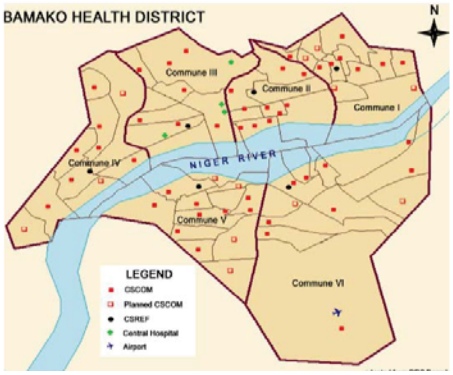

[illegible]enters (a CSRef and one large CSCOM) in rural [illegible]r this approach is that in Mali, rates of mortality [illegible]ildren in rural communities (where the majority of [illegible]amako.

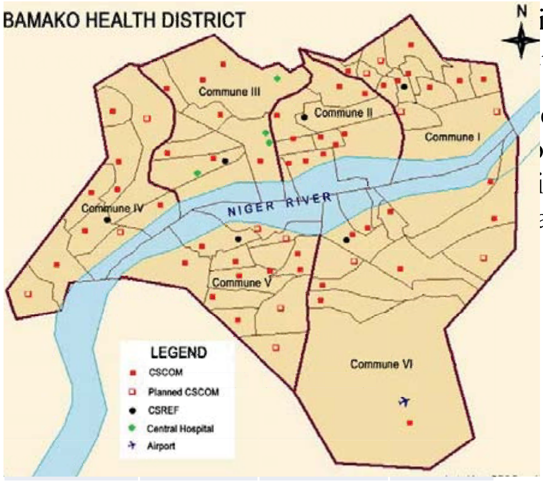

Rural	29	49	
Other regions			
Sikasso	32	48	3.0 million
Kayes	26	38	2.0 million
Koulikoro	26	38	2.4 million
Segou	29	56	2.3 million
Mopti	27	53	2.0 million

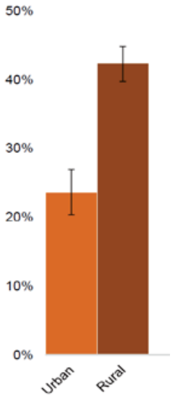

Figure 3. Stunting (<2 SD Ht for age) in Mali

Before the study begins, community sensitization and consent will be obtained. As is the practice for all studies conducted by CVD-Mali investigators, meetings will be convened throughout Bamako and Koulikoro to inform the communities about the study. Religious, political, cultural, and administrative leaders will be invited along with the general community to hear about the purpose, procedures, risks, and benefits of the study. After a question and answer session, the community will render a decision about their willingness to participate. Sensitization and education of health care workers also will be required to establish new practices for appropriate use of antibiotics for children with diarrhoea, as data from GEMS suggest that antibiotics were given or prescribed for the majority of children with MSD.

Brief summary of the implementation strategy

Screening and enrolment visit. We will follow the model we used in GEMS to capture children with diarrhoea (Kotloff 2012, 2013). In brief, study personnel will be situated at the intake area of up to 5 participating health centers. A study registrar will ask the caregiver of all children 2-23 months of age whether the child experienced diarrhoea, according to the study definition. Those with diarrhoea will be referred to the study clinician who will explain the study to the caregiver. The study clinician will determine whether the child meets eligibility criteria. If so, and the caregiver is interested in participation, informed consent will be obtained. The clinician will perform all protocol-specified procedures at the enrolment visit, including: a) measuring height, weight, and MUAC, b) performing a medical history and focused physical examination; c) ensuring that each child receives the recommended therapy for diarrhoea (including rehydration with ORS or intravenous therapy, as appropriate to the level of dehydration, and zinc, according to the IMCI guidelines), d) collecting a stool sample from each enrolled child; e) acquiring the randomization assignment for children who meet all eligibility criteria and whose primary caregiver provides informed consent; f) administering the first dose of study drug/placebo to the child (and all subsequent doses that are indicated while the child remains hospitalized, if applicable); and e) ensuring that procedures are followed to enable relocation of the child for directly observed therapy and follow-up visits.

Relocating participants for home follow-up will be performed with the assistance of community liaisons. In PERCH, cases of severe and very severe pneumonia who lived throughout Bamako were enrolled at HGT. Community liaisons were identified throughout the city. These are literate, nonmedical personnel who are respected in the community. A liaison will be called from the child's community to accompany the child home and accompany the team during follow-up visits. In PERCH, this system resulted in a successful 30-day follow-up visit for >90% of subjects.

Directly observed therapy. DOT will be performed according to the procedures described above in section 5. Serious adverse events will be collected as described in Section 7.

Follow-up visits. Field workers will perform protocol-designated follow-up visits as described in section 6 above. If a child has died on or before day 90, arrangements will be made for the CVD-Mali DSS team to perform a verbal autopsy.

The study protocol and associated research instruments will be submitted to The Centre for Vaccine Development Ethical Review Board (Mali) and the Ethical Review Board of the Maryland University (USA) for review and approval prior to initiating the study.

13.6 Pakistan

Study Area

This study will be conducted in 8 contiguous low income communities among which 4 sites are located approximately 20 km outside Karachi where the Aga Khan University's Department of Pediatrics and Child Health has maintained demographic surveillance since 2003. These communities are coastal settlements, with a total area of 8.1 square miles under surveillance.

Each community has a primary healthcare clinic (PHC) run by staff from the Department of Pediatrics and Child Health, Aga Khan University. These clinics also function as study sites for various research projects. They provide free primary health care services between 9am to 4 pm daily for all children < 5 years of age residing in the demographic surveillance area. These centers also provide vaccination services as per the Expanded Program of Immunization (EPI) in liaison with the local town health officer who provides the EPI vaccines. Children requiring more specialized care are given free transport to a major public sector children's hospital in Karachi. The other two sites are public sector hospitals which are located in close proximity of the two AKU PHC. These public hospitals are working in collaboration with NGO and they are providing health care services to the low income setting communities.

The population according to our last census in these communities is approximately 306,041 with children under five forming almost 14% (44,273) of the total population. The annual birth cohort of these areas is approximately 10,000 The neonatal mortality rate is 45.2 per 1000 live births and the U5MR in these areas is 74.3 per 1000 live births The weighted annual incidence of MSD determined from the GEMS study was 24.5 (12.2-36.8), for the 0-11 months age group and 16.6 (7.3–25.9) and 2.2 (0.8–3.6) per 100 child years for the 12-23 months and 24-59 months age groups respectively. Almost 25% of the children <5 years living in these communities have moderate malnutrition (MUAC <12.5cm). Overall mortality associated with diarrhoeal diseases is estimated to be 1.3%.

The remaining two sites are run by a non-profit organization (Sina Trust Foundation) which provides primary healthcare services to less-privileged communities. Sina clinics are placed across Karachi and more than 80% of Sina patients are women and children They provide services to the patients at their doorstep at a very affordable cost. In total 10 clinics of Sina Trust are functioning in various urban slums across Karachi, 2 of which will be part of this trial

Recruitment

Children will be enrolled from the Aga Khan and Sina managed primary health centers (PHC) and ,nearby local hospitals. Each site will have a study medical physician, research assistant

and community health workers. Every child aged between 2-24 months coming to the centers with diarrhoea will be assessed for eligibility.

At the local hospitals the physician designated for the study will work closely with the hospital staff to identify diarrhoeal children including the outpatient and the admitted children. The community health workers will also work closely with the local area physicians who will be asked to refer diarrhoea patients to the local primary health care center.

Screening/Enrolment

After a potentially eligible child has been identified he/she will be screened for eligibility according to study protocol. If found eligible,the study physician will explain all the study procedures to the primary caregiver. Study details including randomization, drug therapy, chance of receiving placebo, home visits for directly observed therapy (DOT) and follow ups at 45 and 90 days will be explained in detail. If the caregiver is willing to participate the consent will be taken. Consenting will be conducted in the local language (Urdu, Sindhi). Informed consent will be taken in the presence of witnesses. If the caregiver is unable to read and write and the physician will explain the consent in the presence of a community health worker who is not part of the study. The primary caregiver will sign or provide a thumbprint (if illiterate) on the informed consent prior to enrolment.

A stool sample will be collected and processed according to study procedures. All efforts will be made to collect information regarding phone numbers of the caregiver and someone else in the household. This will be used for adherence assessment, follow-up visit reminders, and home tracing if the family moves during the time the child is enrolled in the study.

Following consent, each participant will be assigned a unique study Patient Identification Number (PID). A card detailing the PID and the study site will be given to the primary caregiver of enrolled children. The patient information will be recorded locally by the Research assistants in registers and in the site data base. All registers and CRFs will be stored in a locked file cabinet at each site accessible by only the study clinician and follow-up staff for the purpose of patient tracing. The database will be password protected.

Standard of Care

All enrolled children will receive standard of care for diarrhoea management per WHO and National Health guidelines.

In children with acute diarrhoea, this will include:

a. Standardized assessment of hydration status

b. Rehydration and maintenance fluids with intravenous or oral rehydration solutions (ORS) as indicated

c. Oral zinc therapy for 10-14 days (10 mg for infants < 6 months old; 20 mg for 6 months and older)

d. Recommendation for increased fluid intake and continued feeding at home

e. Instructions on when to seek follow-up care.

Intervention

Following consent and stool sample collection, the study physician will identify the pre-labeled intervention bottle corresponding to the PID. The study physician will explain the regimen and plan for direct observation, directly observe administration of the first dose, and will provide information-motivation-behavior (IMB) theory based counseling on the importance of adhering to the intervention. Direct observation of first dose of study medication will be performed by study staff at the enrolment site. In addition, direct observation of the doses on day 2 and day 3 will be conducted by the mobile team (community health works) in the household on day two and day three. At the end of each day at the enrolment centers, the assigned member of the mobile study team will work with the facility-based study team to determine the visitation schedule for patients enrolled earlier that day.

Follow-up

Between 40 and 50 days since enrolment, a follow-up will be conducted to establish contact and ascertain vital status. The preferred method of contact for the 45-day follow-up will be mobile phone. If no telephone number is provided, or if the participant cannot be reached after four attempts, the mobile study team will conduct a home visit. A standardized script and brief questionnaire will be used to ascertain vital status and to remind the caregiver of the 90-day follow-up visit.

All enrolled children and primary caregivers will be scheduled to return to the health facility at 90 days (between 80 and 100) following enrolment to ascertain vital status and anthropometric measurements. Text message reminders or call to the phone number provided will be done 2 and 1 day prior to the scheduled visit. During this visit, anthropometric measurements will be obtained by the study. Transportation cost will be reimbursed at the follow-up visit thereby incentivizing participants to return. If the participant does not return at their scheduled time, study staff will attempt to make contact with the primary caregiver via cell phone; if no telephone number is provided, or if the participant cannot be reached, study staff will trace the child to the household and bring the caregiver and child to the enrolment center. If the caregiver is not willing to travel to the center, the follow up will be conducted at home If at any follow-up, a caregiver reports that a child has died, study staff trained in verbal autopsy will review hospital records (if available) and will conduct a standardized verbal autopsy at the household to ascertain the cause of death.

Data Management

All data will be collected in real-time using an offline computer-automated questionnaire installed on netbooks at the sites. At the end of each study day, the site staff will sync the offline version of the database with an online secure SQL server. The database is password-protected and all site staff responsible for entering data will have unique usernames and passwords that are controlled by the data manager in Pakistan and will only have rights to enter data. The data manager will evaluate data daily and generates reports on a bi-weekly basis to identify any missing or erroneous data. Changes to existing data will be tracked using an electronic auditing system and adverse events will be recorded in the database.

The study protocol and associated research instruments will be submitted to The Aga Khan University Ethical Review Board for review and approval prior to initiating the study.

References

1. Kotloff KL, Nataro JP, Blackwelder WC, et al. Burden and aetiology of diarrhoeal disease in infants and young children in developing countries (the Global Enteric Multicenter Study, GEMS): a prospective, case-control study. Lancet. 2013;382(9888):209-222.

2. Pavlinac PB, John-Stewart GC, Naulikha JM, et al. High-risk enteric pathogens associated with HIV infection and HIV exposure in Kenyan children with acute diarrhoea. Aids. 2014;28(15):2287-2296.

3. Pavlinac PB, Denno DM, John-Stewart GC, Onchiri FM, Naulikha JM, Odundo EA, Hulseberg CE, Singa BO, Manhart LE, Walson JL. Accuracy of the Integrated Management of Childhood Illness (IMCI) algorithm in identifying culture-confirmed diarrhoeal pathogens requiring antibiotic therapy. Abstract No 1591, 63rd American Society Tropical Medicine and Hygiene Conference, New Orleans, LA, November 2014 (Oral presentation).

4. World Health Organization. Chart Booklet: Integrated Management of Childhood Illness. Geneva, Switzerland. WHO 2014.

13.7 Tanzania

Study Area

The study will take place in peri-urban Dar es Salaam, Tanzania, with a total population of approximately 4.6 million inhabitants. The burden of diarrhoeal disease is high in Tanzania, with an estimated 9% of all deaths in children under age 5 due to diarrhoea [1]. In a recent study using data from verbal autopsies as part of the DHS survey covering 222,958 persons 280 childhood deaths due to diarrhoea were recorded. Forty-eight (60%) of these were due to acute watery diarrhoea and 19 (24%) were due to persistent diarrhoea[2]. In a study to identifydiarrhoeagenic Escherichia coli (DEC) isolated from hospitalized infants and children in Dar es Salaam, it was found that 22.9% of the 280 children had DEC, with the majority being enteroadherent and enteropathogenic E. coli [3]. The effectiveness of IMCI-recommended antibiotics was also examined. More than 90% of the DEC and Shigella sp. were resistant to co-trimoxazole, but all were sensitive to ciprofloxacin [4]. In two recent trials conducted in Dar es Salaam, all-cause mortality rate was 2% in 2400 infants born to HIV-negative mothers and 11% in 2387 infants born to HIV-positive mothers [5].

Recruitment

For this large, randomized, clinical trial, subjects will be recruited from two busy outpatient clinics (Mbagala Rangi Tatu, Mbagala Roundtable) and one large district hospital (Temeke District Hospital). Children with diarrhoea are referred from numerous urban and semi-urban health facilities throughout Dar es Salaam. Prior to the study start, Drs. Manji and Duggan have visited these three sites and described the rationale and logistics of the trial to the hospital leadership and staff. Our preliminary communication with these physicians has confirmed a

high degree of interest in the study, as well as access to patient treatment areas with dedicated space.

Additional recruitment sites may also be used, depending on patient enrolment rates. These include:

1. Main Site (noted above) - Temeke Distric Hospital, / Satellite = Mbagala Rangi Tatu, Mbagala Round table Yombo Vituka and Yombo Reli ,Vingunguti, Kiwalani, Taima Akudo
2. Main Site - Amana Hospital / Satellite =, AKC CTC , Mnazi Mmoja , Gerezani, TRC Dispensary
3. Main Site - Mwananyamala Hospital / Satellite = Sinza, Tandale, Magomeni, , Kimara, Mwenge

Research nurses will be placed at each of the three selected sites for recruitment. Study staff will work closely with hospital staff to determine when there is a potentially eligible child either on the ward or in the outpatient unit, a system that has been successfully used to recruit children in our past clinical trials. Additionally, a team focused solely on community sensitization, directly observed therapy, and follow-up (mobile team) will be deployed into the community, enriching enrolment by working with existing Ministry of Health community health clinics that commonly refer patients to one of the enrolment hospitals. Each enrolment center will be assigned a mobile team member who will travel to participants' homes (see below).

Screening/Enrolment

After a potentially eligible child has been identified, confirmation of all inclusion and exclusion criteria will be performed by the study nurse, and will include physical examination of children by study physicians to assess degree of dehydration, as well as to confirm the absence of any contraindication to study participation. These will include any IMCI general danger signs (e.g., unable able to drink or breastfeed, convulsions, vomiting, unconscious, stiff neck) or evidence of another illness requiring antibiotics (e.g., pneumonia) or other drugs that are contraindicated (e.g., coartem).

If no contraindication to study participation is found, a study nurse will approach the primary caregiver of the child and describe the study procedures. Study staff will explain that the child and caregiver must be willing to be visited at the home for directly observed therapy (DOT) for the next two days, be willing to return to the health facility 90-days after enrolment, and be willing to provide a stool from the child at enrolment and possibly at 90 days (for a subset of randomly selected children). If the child is eligible and the caregiver interested in participating, written informed consent will be obtained in Kiswahili. The parent or guardian (primary caregiver) must sign written informed consent (or provide a witnessed thumbprint if not literate) prior to enrolment.

After informed consent is obtained, research procedures will commence. These will include tablet-based questionnaires for data concerning history of diarrhoea (duration, severity, frequency, accompanying vomiting), baseline demographic, socioeconomic and past medical history, breastfeeding and vaccination history, and the HIV status of the primary caregiver and child (if known) will be recorded. A stool sample will be collected in a sterile stool container and processed according to study procedures.

The follow-up research nurse will collect detailed contact information from the caregivers using a standardized patient locator form, including at least two working cell phone numbers for the caregiver, as well as a physical description of the whereabouts of the child's home (so-called "mapcues"). If a caregiver does not have a personal mobile phone we will obtain the mobile phone information of a household member or friend of the caregivers' choosing. We expect fewer than 5% of participants to be without cell phone access based on our past diarrhoea studies.

Standard of Care

All enrolled children will receive standard of care for diarrhoea management per WHO and Tanzanian Ministry of Health guidelines.

In children with acute diarrhoea, this will include:

a. Standardized assessment of hydration status

b. Rehydration and maintenance fluids with intravenous or oral rehydration solutions (ORS) as indicated

c. Oral zinc therapy for 10-14 days (10 mg for infants < 6 months old; 20 mg for 6 months and older)

d. Recommendation for increased fluid intake and continued feeding at home

e. Instructions on when to seek follow-up care.

Intervention

Following consent and stool sample collection, the study nurse will identify the pre-labeled intervention bottle corresponding to the patient ID number. The study nurse will explain the regimen and plan for direct observation, directly observe administration of the first dose, and will provide information-motivation-behavior (IMB) theory based counseling on the importance of adhering to the intervention. Direct observation of first dose of study medication will be performed by study staff at the enrolment site. In addition, direct observation of the doses on day 2 and day 3 will be conducted by the mobile team in the household on day two and day three. At the end of each day at the enrolment centers, the assigned member of the mobile study team will work with the facility-based study team to determine the visitation schedule for patients enrolled earlier that day and walk through the patient locator form with the counselor.

Follow-up

Between 40 and 50 days since enrolment, a follow-up will be conducted to establish contact and ascertain vital status. The preferred method of contact for the 45-day follow-up will be mobile phone. If no telephone number is provided, or if the participant cannot be reached after four attempts, the mobile study team will conduct a home visit. A standardized script and brief questionnaire will be used to ascertain vital status and to remind the caregiver of the 90-day follow-up visit.

All enrolled children and primary caregivers will be scheduled to return to the health facility at 90 days (between 80 and 100) following enrolment to ascertain vital status and obtain anthropometric measurements. Text message reminders will be sent to the caregiver's phone number 2 and 1 days prior to the scheduled visit. During this visit, anthropometric measurements will be obtained by a research nurse, and caregivers will be asked about any hospitalizations or other severe adverse event (SAE) occurring since the last time the child was seen by study staff. Transportation cost will be reimbursed at the follow-up visit thereby incentivizing participants to return. If the participant does not return at their scheduled time, study staff will attempt to make contact with the primary caregiver via cell phone; if no telephone number is provided, or if the participant cannot be reached, study staff will trace the child to the household and bring the caregiver and child to the enrolment hospital. If at any follow-up, a caregiver reports that a child has died, study staff trained in verbal autopsy will review hospital records (if available) and will conduct a standardized verbal autopsy at the household to ascertain the cause of death.

Data Management

All data will be collected in real-time using an offline computer-automated questionnaire installed on netbooks at the sites. At the end of each study day, the site staff will sync the offline version of the database with an online secure SQL server. The database is password-protected and all site staff responsible for entering data will have unique usernames and passwords that are controlled by the data manager and will only have rights to enter data. The MUHAS-based data manager evaluates data daily and generates reports on a weekly basis to identify any missing or erroneous data. On a monthly basis, the Geneva-based data manager will cross-check the data being entered by generating a detailed monthly report. Changes to existing data will be tracked using an electronic auditing system and adverse events will be recorded in the database.

The study protocol and associated research instruments have been submitted to Muhimbili University Ethical Review Board (Tanzania) and the IRB of Boston Children's Hospital for review.

References

1. Liu L, Johnson HL, Cousens S, et al. Global, regional, and national causes of child mortality: an updated systematic analysis for 2010 with time trends since 2000. The Lancet. 2012;379:2151-2161.

2. Rahman AE, Moinuddin M, Molla M, et al. Childhood diarrhoeal deaths in seven low- and middle-income countries. Bull World Health Organ. 2014;92(9):664-671.

3. Moyo S, Maselle S, Matee M, Langeland N, Mylvaganam H. Identification of diarrhoeagenic Escherichia coli isolated from infants and children in Dar es Salaam, Tanzania. BMC Infectious Diseases. 2007;7(1):92.

4. Moyo SJ, Gro N, Matee MI, et al. Age specific aetiological agents of diarrhoea in hospitalized children aged less than five years in Dar es Salaam, Tanzania. BMC Pediatr. 2011;11:19.

5. Duggan C, Manji KP, Kupka R, et al. Multiple micronutrient supplementation in Tanzanian infants born to HIV-infected mothers: a randomized, double-blind, placebo-controlled clinical trial. Am J Clin Nutr. 2012;96(6):1437-1446.
