# Supplementary material for: Effect of 3 Days of Oral Azithromycin on Young Children With Acute Diarrhea in Low-Resource Settings: A Randomized Clinical Trial
Source: JAMA Netw Open. 2021 Dec 16;4(12):e2136726. doi: 10.1001/jamanetworkopen.2021.36726 (PMC8678692; doi:10.1001/jamanetworkopen.2021.36726)
Supplement: Supplement 2. — eTable 1. Cause of Death Among Children in the ABCD Trial eTable 2. Per Protocol Analyses of Primary Outcomes eTable 3. List of Recruiting Centres eTable 4. Proportion of E. coli Isolates From Index Children With Antibiotic Resistance by Randomization Arm and Timepoint eTable 5. Proportion of S. pneumoniae Isolates From Index Children With Antibiotic Resistance by Randomization Arm and Timepoint eTable 6. Proportion of E. coli Isolates From Child Contacts With Antibiotic Resistance by Randomization Arm and Timepoint eTable 7. Proportion of S. pneumoniae Isolates From Child Contacts With Antibiotic Resistance by Randomization Arm and Timepoint eFigure 1. Effect Modification on the Primary Outcome (Mortality) in Different Strata (Point Estimates and 95% CI) by a Priori Defined Effect Modifiers eFigure 2. Effect Modification on the Primary Outcome (Change in Linear Growth) in Different Strata (Point Estimates and 95% CI) by a Priori Defined Effect Modifiers [file jamanetwopen-e2136726-s002.pdf]

Supplementary Online Content

The Antibiotics for Children With Diarrhea (ABCD) Study Group. Effect of 3 days of oral azithromycin on young children with acute diarrhea in low-resource settings: a randomized clinical trial. *JAMA Netw Open.* 2021;4(12):e2136726. doi:10.1001/jamanetworkopen.2021.36726

eTable 1. Cause of Death Among Children in the ABCD Trial
eTable 2. Per Protocol Analyses of Primary Outcomes
eTable 3. List of Recruiting Centres
eTable 4. Proportion of *E. coli* Isolates From Index Children With Antibiotic Resistance by Randomization Arm and Timepoint
eTable 5. Proportion of *S. pneumoniae* Isolates From Index Children With Antibiotic Resistance by Randomization Arm and Timepoint
eTable 6. Proportion of *E. coli* Isolates From Child Contacts With Antibiotic Resistance by Randomization Arm and Timepoint
eTable 7. Proportion of *S. pneumoniae* Isolates From Child Contacts With Antibiotic Resistance by Randomization Arm and Timepoint
eFigure 1. Effect Modification on the Primary Outcome (Mortality) in Different Strata (Point Estimates and 95% CI) by a Priori Defined Effect Modifiers
eFigure 2. Effect Modification on the Primary Outcome (Change in Linear Growth) in Different Strata (Point Estimates and 95% CI) by a Priori Defined Effect Modifiers

This supplementary material has been provided by the authors to give readers additional information about their work.

© 2021 The Antibiotics for Children With Diarrhea (ABCD) Study Group. *JAMA Network Open.*

eTable 1. Cause of Death Among Children in the ABCD Trial

**Cause of death**	**Placebo**	**Azithromycin**	**Total**
**Infections**	18	12	30
Diarrhoea	*6*	*3*	*9*
Sepsis	*5*	*4*	*9*
Pneumonia	*5*	*2*	*7*
Meningitis	*1*	*2*	*3*
Malaria	*1*	*0*	*1*
Measles	*0*	*1*	*1*
**Accidents**	3	5	8
**Severe malnutrition**	3	0	3
**Other***	3	1	4
**Indeterminate**	1	2	3
**Total**	28	20	48

**includes acute abdomen, HIV related, asthma, myocarditis*

© 2021 The Antibiotics for Children With Diarrhea (ABCD) Study Group. *JAMA Network Open.*

eTable 2. Per Protocol# Analyses of Primary Outcomes

Outcome	Placebo	Azithromycin	Effect (95% CI)	P-value
180-day mortality, no. (%)<br>(Participants analysed, n)	28 (0.68)<br>(n=4024)	19 (0.47)<br>(n=4038)	RR 0.68 (0.38, 1.21)*	0.25
90 day ΔLAZ, mean change (SD)<br>(Participants analysed, n)	–0.19 (0.60)<br>(n=3871)	–0.15 (0.59)<br>(n=3874)	RD 0.03 (0.01, 0.06) †	0.007

# For both outcomes, this analysis excluded participants who were not adherent to the treatment or were ineligible but enrolled. For the ΔLAZ outcome, the same participants were excluded in addition to those who had a missing outcome data for ΔLAZ

** Risk ratio(RR) from log-binomial regression, adjusted for country*

† Risk difference(RD) in ΔLAZ from linear regression adjusted for baseline LAZ and country, standard errors account for clustering by participant

SD = standard deviation; CI = confidence interval; ΔLAZ = mean change in length-for-age Z-score

© 2021 The Antibiotics for Children With Diarrhea (ABCD) Study Group. *JAMA Network Open.*

eTable 3. List of Recruiting Centres

**Recruiting centre**	**Type of facility**	**Rural/Urban**
Country: BANGLADESH		
Dhaka Hospital, International Centre for Diarrheal Disease Research, Bangladesh (icddr,b)	Hospital Triage/ Short Stay Ward	Urban
Mirpur Treatment Centre, International Centre for Diarrheal Disease Research, Bangladesh (icddr,b)	Hospital Triage/ Short Stay Ward	Urban
Country: INDIA		
P L Sharma District Hospital	Hospital out-patient department	Urban
Lala Lajapat Rai Memorial Medical College	Hospital out-patient department	Both- Rural and Urban
Community health center Mawana	Community health center	Rural
Community health center Sardhana	Community health center	Rural
Country: KENYA		
Awendo Sub-County Hospital	Hospital- Outpatient and Inpatient	Urban
Isebania Sub-County Hospital	Hospital- Outpatient and Inpatient	Urban
Mbita sub-County Hospital	Hospital- Outpatient and Inpatient	Urban
Ndhiwa Sub-County Hospital	Hospital- Outpatient and Inpatient	Urban
Rachuonyo Sub-County Hospital	Hospital- Outpatient and Inpatient	Urban
Rongo Sub-County Hospital	Hospital- Outpatient and Inpatient	Urban
Country: MALI		
l'Hôpital Gabriel Touré	Hospital	Urban
CSREF Commune V	Referral Health Center	Urban
CSREF Koulikoro	Referral Health Center	Rural
CSCOM Koulikoro	Community Health Center	Rural
CSREF Commune 6	Referral Health Center	Urban
CSREF Commune 4	Referral Health Center	Urban
CSREF Commune 1	Referral Health Center	Urban
CSREF Kalabancoro	Referral Health Center	Urban
Country: MALAWI		
Bangwe Health Center	Out-patient under 5 clinic	Urban
Limbe Health Center	Out-patient under 5 clinic	Urban

© 2021 The Antibiotics for Children With Diarrhea (ABCD) Study Group. *JAMA Network Open.*

Ndirande Health Center	Out-patient under 5 clinic	Urban
Chilomoni Health Center	Out-patient under 5 clinic	Urban
Mbayani Health Center	Out-patient under 5 clinic	Urban
Queen Elizabeth Central Hospital	Hospital clinic	Urban
Country: PAKISTAN		
Ali Akbar Shah	AKU Primary health center	Peri Urban
Ibrahim Hyderi	AKU Primary health center	Peri Urban
Bhains Colony	AKU Primary health center	Peri Urban
Shireen Jinnah Colony	Sina NGO Primary health center	Peri Urban
Machar Colony	Sina NGO Primary health center	Peri Urban
Sindhi Govt Hospital Korangin No. 5	Hospital	Peri Urban
Sindhi Govt Hospital Ibrahim Hyderi	Hospital	Peri Urban
Country: TANZANIA		
Temeke Regional Referral Hospital	Outpatient clinic of Pediatrics	Semi-urban
Mbagala RangiTatu	Outpatient Pediatric Clinic	Semi-Urban
Mbagala Round Table	Outpatient Pediatric Clinic	Rural

© 2021 The Antibiotics for Children With Diarrhea (ABCD) Study Group. *JAMA Network Open.*

eTable 4. Proportion of *E. coli* Isolates From Index Children With Antibiotic Resistance by Randomization Arm and Timepoint

	Day 1				Day 90				Day 180			
	**Azithromycin-arm (n=829)**		**Placebo-arm (n=840)**		**Azithromycin-arm (n=738)**		**Placebo-arm (n=758)**		**Azithromycin-arm (n=672)**		**Placebo-arm (n=713)**	
	%	95% CI*	%	95% CI*	%	95% CI*	%	95% CI*	%	95% CI*	%	95% CI*
**Ampicillin**	89.6	(87.3, 91.6)	91.2	(89.1, 93)	85.2	(82.4, 87.7)	84.7	(81.9, 87.2)	85.2	(82.4, 87.7)	78.7	(75.5, 81.7)
**Amoxicillin/ K Clavulanate**	39.4	(36.1, 42.9)	37.5	(34.2, 40.9)	36.5	(33.0, 40.1)	39.1	(35.6, 42.6)	36.5	(33.0, 40.1)	35.7	(32.2, 39.4)
**Ceftazidime**	42.3	(38.9, 45.8)	41.8	(38.4, 45.2)	42.1	(38.5, 45.7)	46.7	(43.1, 50.3)	42.1	(38.5, 45.7)	42.0	(38.4, 45.7)
**Cefuroxime**	45.0	(41.6, 48.5)	44.2	(40.8, 47.6)	45.3	(41.7, 49.0)	49.7	(46.1, 53.4)	45.3	(41.7, 49.0)	47.1	(43.3, 50.8)
**Imipenem**	10.1	(8.2, 12.4)	9.3	(7.4, 11.5)	9.1	(7.1, 11.4)	9.0	(7, 11.2)	9.1	(7.1, 11.4)	8.1	(6.2, 10.4)
**Meropenem**	3.7	(2.6, 5.3)	2.4	(1.5, 3.7)	5.3	(3.8, 7.2)	5.3	(3.8, 7.1)	5.3	(3.8, 7.2)	6.4	(4.8, 8.5)
**Amikacin**	1.2	(0.6, 2.2)	1.5	(0.8, 2.6)	1.5	(0.7, 2.7)	0.9	(0.4, 1.9)	1.5	(0.7, 2.7)	1.3	(0.6, 2.4)
**Trimethoprim/ Sulfamethoxazole**	78.4	(75.4, 81.2)	78.7	(75.8, 81.4)	73.4	(70.1, 76.6)	71.0	(67.6, 74.2)	73.4	(70.1, 76.6)	70.4	(67, 73.8)
**Chloramphenicol**	15.9	(13.5, 18.6)	20.5	(17.8, 23.4)	16.7	(14.1, 19.6)	15.8	(13.3, 18.6)	16.7	(14.1, 19.6)	14.1	(11.7, 16.9)
**Tetracycline**	70.1	(66.8, 73.2)	69.5	(66.3, 72.6)	60.7	(57.0, 64.2)	59.9	(56.3, 63.4)	60.7	(57.0, 64.2)	59.8	(56.1, 63.4)
**Multi-drug resistant†**	62.4	(59.0-65.7)	61.0	(57.7-64.4)	59.9	(56.3, 63.4)	62.7	(59.2, 66.2)	59.9	(56.3, 63.4)	55.5	(51.7, 59.1)

* *Assuming a binomial distribution*

†Defined as resistant to one or more antibiotics within 3 or more classes (aminoglycosides, carbapenem, cephem, fluroquinolone, folate pathway inhibitor, fosfomycin, macrolide, monobactam, penicillin, phenicol, tetracycline)

© 2021 The Antibiotics for Children With Diarrhea (ABCD) Study Group. *JAMA Network Open.*

eTable 5. Proportion of *S. pneumoniae* Isolates From Index Children With Antibiotic Resistance by Randomization Arm and Timepoint

	Day 90						Day 180			
	Azithromycin-arm (n=543)		Placebo-arm (n=562)				Azithromycin-arm (n=477)		Placebo-arm (n=522)	
	%	95% CI*	%	95% CI*			%	95% CI*	%	95% CI*
Penicillin	59.1	(54.8, 63.3)	61.9	(57.8, 66)			60.4	(55.8, 64.8)	65.3	(61.1, 69.4)
Amoxicillin/Clavulanate	2.6	(1.4, 4.3)	2.3	(1.2, 3.9)			3.6	(2.1, 5.6)	3.8	(2.4, 5.9)
Ceftriaxone	5.9	(4.1, 8.2)	4.8	(3.2, 6.9)			7.3	(5.2, 10.1)	6.3	(4.4, 8.8)
Meropenem	9.0	(6.8, 11.8)	7.5	(5.4, 10)			8.8	(6.4, 11.7)	10.0	(7.5, 12.9)
Levofloxacin	4.8	(3.2, 6.9)	5.5	(3.8, 7.7)			4.4	(2.7, 6.7)	4.8	(3.1, 7)
Clarithromycin	42.4	(38.2, 46.6)	38.8	(34.7, 43)			43.4	(38.9, 48)	39.8	(35.6, 44.2)
Erythromycin	45.7	(41.4, 50.0)	42.3	(38.2, 46.6)			45.7	(41.1, 50.3)	41.0	(36.7, 45.3)
Chloramphenicol	9.2	(6.9, 12.0)	6.2	(4.4, 8.6)			8.4	(6.1, 11.2)	7.3	(5.2, 9.9)
Tetracycline	54.7	(50.4, 58.9)	52.3	(48.1, 56.5)			52.2	(47.6, 56.8)	54.8	(50.4, 59.1)
Multi-drug resistance[†]	59.7	(55.4, 63.8)	60.7	(56.5, 64.7)			62.3	(57.7, 66.6)	62.5	(58.1, 66.6)

* Assuming a binomial distribution
† Defined as resistant to one or more antibiotics within 3 or more classes (ansamycins, carbapenem, cephem, fluroquinolone, folate pathway inhibitor, glycopeptide, lincosamide, macrolide, penicillin, phenicol, streptomycin, tetracyclines)

© 2021 The Antibiotics for Children With Diarrhea (ABCD) Study Group. *JAMA Network Open.*

eTable 6. Proportion of *E. coli* Isolates From Child Contacts With Antibiotic Resistance by Randomization Arm and Timepoint

	Day 90				Day 180			
	**Azithromycin-arm (n=338)**		**Placebo-arm (n=349)**		**Azithromycin-arm (n=302)**		**Placebo-arm (n=325)**	
	%	95% CI*	%	95% CI*	%	95% CI*	%	95% CI*
**Ampicillin**	63.4	(56.8, 69.6)	64.6	(58.2, 70.6)	54.7	(47.6, 61.7)	62.9	(56.3, 69.2)
**Amoxicillin/ K Clavulanate**	2.2	(0.7, 5)	4.6	(2.3, 8)	4.5	(2.1, 8.4)	4.8	(2.4, 8.4)
**Ceftazidime**	11.6	(7.8, 16.5)	10.4	(6.8, 14.9)	8.5	(5, 13.2)	5.7	(3.1, 9.5)
**Cefuroxime**	7.8	(4.7, 12)	7.5	(4.5, 11.6)	4.5	(2.1, 8.5)	7.4	(4.4, 11.6)
**Imipenem**	37.9	(31.7, 44.5)	32.8	(26.9, 39.1)	34.5	(27.9, 41.5)	33.2	(27.1, 39.7)
**Meropenem**	23.9	(18.4, 30)	14.7	(10.4, 20)	14.1	(9.6, 19.7)	13.3	(9.2, 18.5)
**Amikacin**	36.6	(30.4, 43.2)	34	(28.1, 40.4)	34.5	(27.9, 41.5)	32.5	(26.4, 39)
**Trimethoprim/ Sulfamethoxazole**	37.5	(31.3, 44.1)	33.6	(27.7, 40)	31.3	(25, 38.2)	32.8	(26.7, 39.2)
**Chloramphenicol**	9.1	(5.7, 13.6)	7.1	(4.2, 11.1)	8	(4.6, 12.6)	4.8	(2.4, 8.4)
**Tetracycline**	45.7	(39.2, 52.3)	49.4	(42.9, 55.9)	49.8	(42.6, 56.9)	48.9	(42.3, 55.6)
**Multi-drug resistance†**	56	(49.4, 62.5)	55.2	(48.7, 61.6)	51.2	(44.1, 58.3)	53.7	(47, 60.3)

*** Assuming a binomial distribution**

†Defined as resistant to one or more antibiotics within 3 or more classes (aminoglycosides, carbapenem, cephem, fluroquinolone, folate pathway inhibitor, fosfomycin, macrolide, monobactam, penicillin, phenicol, tetracycline)

© 2021 The Antibiotics for Children With Diarrhea (ABCD) Study Group. *JAMA Network Open.*

eTable 7. Proportion of *S. pneumoniae* Isolates From Child Contacts With Antibiotic Resistance by Randomization Arm and Timepoint

	Day 90				Day 180			
	Azithromycin-arm (n=232)		Placebo-arm (n=241)		Azithromycin-arm (n=201)		Placebo-arm (n=229)	
	%	95% CI	%	95% CI	%	95% CI	%	95% CI
Penicillin	63.4	(56.8, 69.6)	64.6	(58.2, 70.6)	54.7	(47.6, 61.7)	65.3	(61.1, 69.4)
Amoxicillin/Clavulanate	2.2	(0.7, 5)	4.6	(2.3, 8)	4.5	(2.1, 8.4)	3.8	(2.4, 5.9)
Ceftriaxone	11.6	(7.8, 16.5)	10.4	(6.8, 14.9)	8.5	(5, 13.2)	6.3	(4.4, 8.8)
Meropenem	9.1	(5.7, 13.5)	10.8	(7.2, 15.4)	9.5	(5.8, 14.4)	9.6	(6.1, 14.2)
Levofloxacin	7.8	(4.7, 12)	7.5	(4.5, 11.6)	4.5	(2.1, 8.5)	7.4	(4.4, 11.6)
Clarithromycin	37.9	(31.7, 44.5)	32.8	(26.9, 39.1)	34.5	(27.9, 41.5)	33.2	(27.1, 39.7)
Erythromycin	36.6	(30.4, 43.2)	34.0	(28.1, 40.4)	34.5	(27.9, 41.5)	32.5	(26.4, 39)
Chloramphenicol	9.1	(5.7, 13.6)	7.1	(4.2, 11.1)	8.0	(4.6, 12.6)	4.8	(2.4, 8.4)
Tetracycline	45.7	(39.2, 52.3)	49.4	(42.9, 55.9)	49.8	(42.6, 56.9)	48.9	(42.3, 55.6)
Multi-drug resistance†	56.0	(49.4, 62.5)	55.2	(48.7, 61.6)	51.2	(44.1, 58.3)	53.7	(47, 60.3)

* Assuming a binomial distribution

† Defined as resistant to one or more antibiotics within 3 or more classes (ansamycins, carbapenem, cephem, fluroquinolone, folate pathway inhibitor, glycopeptide, lincosamide, macrolide, penicillin, phenicol, streptomycin, tetracyclines)

© 2021 The Antibiotics for Children With Diarrhea (ABCD) Study Group. *JAMA Network Open.*

eFigure 1. Effect Modification on the Primary Outcome (Mortality) in Different Strata (Point Estimates and 95% CI) by a Priori Defined Effect Modifiers

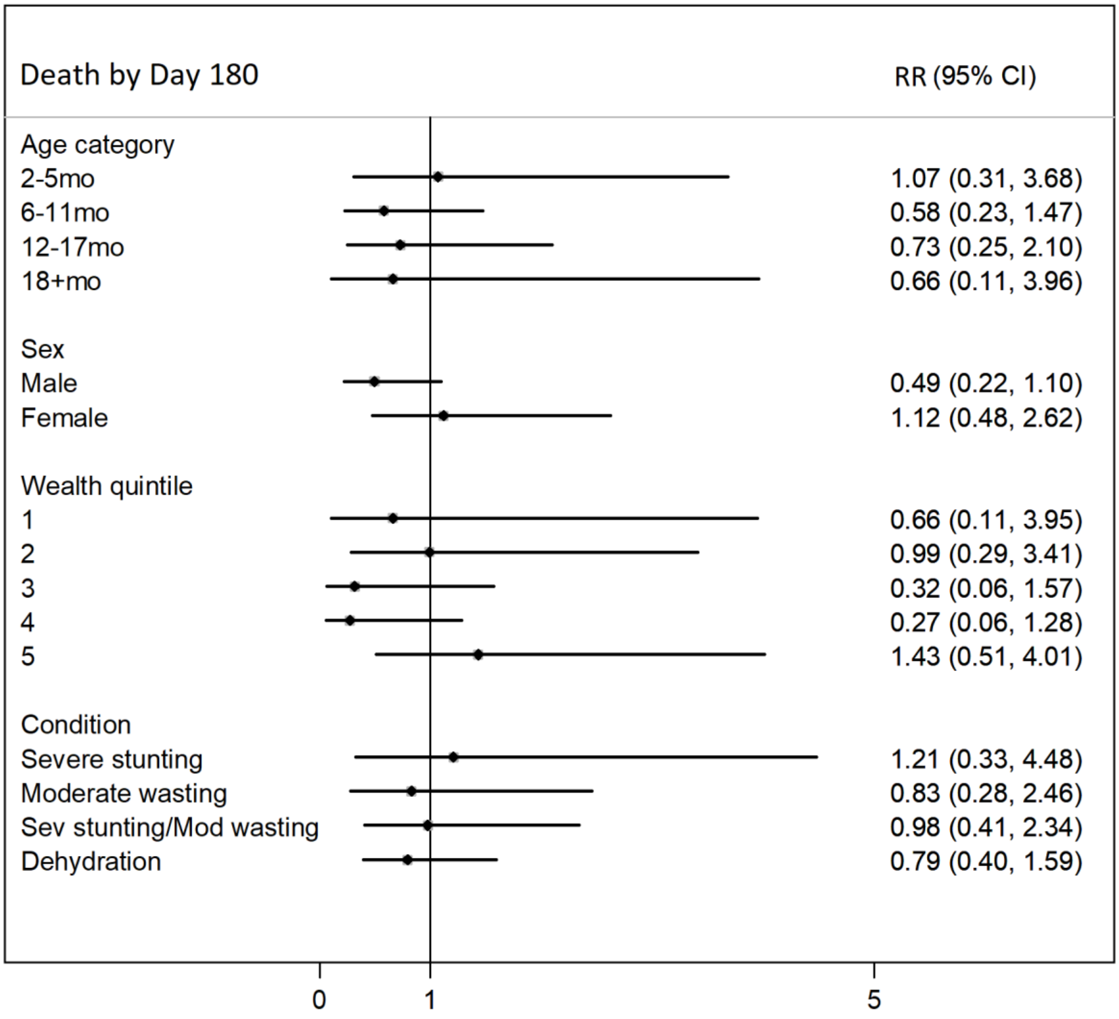

© 2021 The Antibiotics for Children With Diarrhea (ABCD) Study Group. *JAMA Network Open.*

eFigure 2. Effect Modification on the Primary Outcome (Change in Linear Growth) in Different Strata (Point Estimates and 95% CI) by a Priori Defined Effect Modifiers

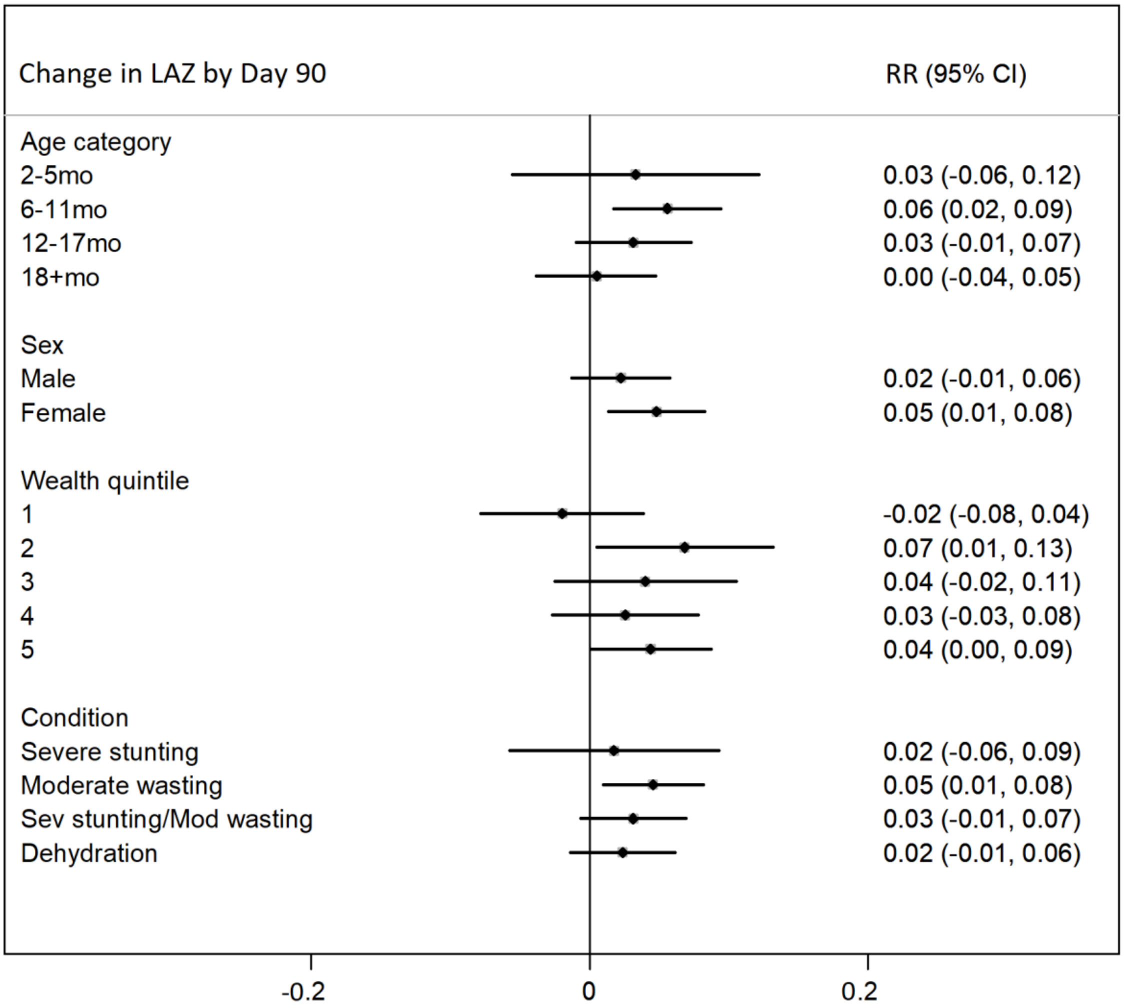

© 2021 The Antibiotics for Children With Diarrhea (ABCD) Study Group. *JAMA Network Open.*
