# Supplementary material for: Effect of 3 Days of Oral Azithromycin on Young Children With Acute Diarrhea in Low-Resource Settings: A Randomized Clinical Trial
Source: JAMA Netw Open. 2021 Dec 16;4(12):e2136726. doi: 10.1001/jamanetworkopen.2021.36726 (PMC8678692; doi:10.1001/jamanetworkopen.2021.36726)
Supplement: Supplement 3. — Data Sharing Statement [file jamanetwopen-e2136726-s003.pdf]

Data Sharing Statement

Ahmed. Effect of 3 Days of Oral Azithromycin on Young Children With Acute Diarrhea in Low-Resource Settings. *JAMA Netw Open*. Published December 16, 2021.
doi:10.1001/jamanetworkopen.2021.36726

Data
Data available: Yes
Data types: Deidentified participant data
How to access data: Request for data from deay@who.int
When available: With publication

Supporting Documents
Document types: None

Additional Information
Who can access the data: Researchers whose proposed used of data has been approved
Types of analyses: Specfied purpose
Mechanisms of data availability: Signed data access agreement
Any additional restrictions: NA
